# Constitutive activation of oncogenic PDGFRα-mutant proteins occurring in GIST patients induces receptor mislocalisation and alters PDGFRα signalling characteristics

**DOI:** 10.1186/s12964-015-0096-8

**Published:** 2015-03-31

**Authors:** Christelle Bahlawane, René Eulenfeld, Monique Y Wiesinger, Jiali Wang, Arnaud Muller, Andreas Girod, Petr V Nazarov, Kathrin Felsch, Laurent Vallar, Thomas Sauter, Venkata P Satagopam, Serge Haan

**Affiliations:** Molecular Disease Mechanisms Group, Life Sciences Research Unit, University of Luxembourg, 162A Avenue de la Faïencerie, L-1511 Luxembourg, Luxembourg; Signal Transduction Group, Life Sciences Research Unit, University of Luxembourg, 162A Avenue de la Faïencerie, L-1511 Luxembourg, Luxembourg; Systems Biology Group, Life Sciences Research Unit, University of Luxembourg, 162A Avenue de la Faïencerie, L-1511 Luxembourg, Luxembourg; Genomics Research Unit, Luxembourg Institute of Health, 84 Val Fleuri, L-1526 Luxembourg, Luxembourg; Light Microscopy Facility, Life Sciences Research Unit, University of Luxembourg, 162a Avenue de la Faïencerie, L-1511 Luxembourg, Luxembourg; Deparment of Biochemistry, RWTH-Aachen University, Pauwelsstr. 30, D-52074 Aachen, Germany; Luxembourg Centre for Systems Biomedicine, University of Luxembourg, 7 Avenue des Hauts-Fourneaux, L-4362 Esch-sur-Alzette, Luxembourg

**Keywords:** PDGFRα, Gastrointestinal stromal tumour, GIST, STAT, AKT, MAPK

## Abstract

**Background:**

Gastrointestinal stromal tumours (GIST) are mainly characterised by the presence of activating mutations in either of the two receptor tyrosine kinases c-KIT or platelet-derived growth factor receptor-α (PDGFRα). Most mechanistic studies dealing with GIST mutations have focused on c-KIT and far less is known about the signalling characteristics of the mutated PDGFRα proteins. Here, we study the signalling capacities and corresponding transcriptional responses of the different PDGFRα proteins under comparable genomic conditions.

**Results:**

We demonstrate that the constitutive signalling via the oncogenic PDGFRα mutants favours a mislocalisation of the receptors and that this modifies the signalling characteristics of the mutated receptors. We show that signalling via the oncogenic PDGFRα mutants is not solely characterised by a constitutive activation of the conventional PDGFRα signalling pathways. In contrast to wild-type PDGFRα signal transduction, the activation of STAT factors (STAT1, STAT3 and STAT5) is an integral part of signalling mediated via mutated PDGF-receptors. Furthermore, this unconventional STAT activation by mutated PDGFRα is already initiated in the endoplasmic reticulum whereas the conventional signalling pathways rather require cell surface expression of the receptor. Finally, we demonstrate that the activation of STAT factors also translates into a biologic response as highlighted by the induction of STAT target genes.

**Conclusion:**

We show that the overall oncogenic response is the result of different signatures emanating from different cellular compartments. Furthermore, STAT mediated responses are an integral part of mutated PDGFRα signalling.

**Electronic supplementary material:**

The online version of this article (doi:10.1186/s12964-015-0096-8) contains supplementary material, which is available to authorized users.

## Background

The prominent role for the platelet derived growth factor (PDGF) in oncogenesis, angiogenesis and tumour metastasis is well known and the excessive activation of the PDGF receptors PDGFRα and PDGFRβ is found in many cancer settings [1-4]. GIST is the most common mesenchymal neoplasm in the gastrointestinal tract and is characterized by the presence of mutations in the kinases KIT or PDGFRα [5,6]. Ligand-independent, constitutively active mutations of PDGFRα occur in about 5-8% of patients [7]. The identified mutations circumvent the autoinhibition of the kinase domain and lead to its constitutive activity [8]. The PDGFRα mutations affect two regions within the receptor, the autoinhibitory juxtamembrane region (exon 12 mutations) and the kinase domain itself (exon 14 and exon 18 mutations). Whereas exon 14 mutations affect the upper lobe of the kinase domain, exon 18 mutations are located within the activation loop [5]. In this study, we investigate the signalling characteristics and transcriptomic responses of mutated PDGFRα proteins and compare them to the wild-type receptor. For this, we selected one point mutation in the juxtamembrane region (V561D in exon 12) and two activation loop point mutations (D842V and D842Y in exon 18).

Gleevec (Imatinib, STI751), a drug originally developed to inhibit the Bcr-Abl fusion protein found in chronic myelogenous leukaemia, also shows substantial inhibitory activity against PDGFRs and KIT and is currently used in the first line therapy for GIST patients. However, about 60-80% of the GIST patients with a mutated PDGFRα carry the Gleevec insensitive mutation D842V [9]. Most interestingly, the D842Y-mutation is sensitive to treatment with Gleevec. Other mutations such as the V561D mutation in the juxtamembrane region of PDGFRα are also Gleevec-sensitive [10]. Patients carrying Gleevec insensitive mutations can be treated with the kinase inhibitor sunitinib but this TKI shows only weak inhibitory activity on PDGFRα-D842V [11]. Another tyrosine kinase inhibitor, dasatinib, was shown to inhibit the D842V mutant [12] and has recently been subject to clinical trials in the context of GIST treatment.

Ligand-activated PDGFRα wild-type recruits intracellular signalling molecules mostly via their SH2 domains to the phosphorylated tyrosine motifs within the receptor. The subsequent activation of these signalling components (e.g. phospholipase Cγ (PLCγ), phosphatidylinositol 3-kinase (PI3K), Src kinases and others leads to the transcription of target genes and the cellular response [3,4].

Signal transducer and activator of transcription (STAT) proteins are transcription factors that classically mediate cytokine responses. In recent years their importance for carcinogenesis and tumour associated inflammation became clear [13-15]. Numerous studies have reported the activation of STAT1, STAT3 and STAT5 in hematologic malignancies and solid tumours. The effects of STAT factor activation on tumourigenesis can be very diverse and largely depend on the kind of tumour and its microenvironment. STAT factors can either inhibit (mostly STAT1) or support (mostly STAT3 and STAT5) tumour cell proliferation. In addition, they have profound effects on the tumour microenvironment and tumour associated inflammation.

In this study, we set out to compare the signalling capacities and transcriptional responses of different oncogenic PDGFRα mutants occurring in GIST patients with those of the wild-type PDGF-receptor. In addition, we wanted to assess whether the location of the mutation, either membrane-proximal or within the activation loop of the kinase causes differences in signalling and cellular responses. In order to investigate and compare the signalling characteristics and the corresponding biologic responses of different PDGFRα mutant proteins, we generated stable cell lines that allow inducible expression of wild-type PDGFRα and PDGFRα mutant proteins on an isogenic background. Comparative studies of the signal transduction capacities of different mutant proteins cannot be performed in patient cells due to genetic variability and the high genetic instability of cancer cells. These differences may influence the signalling behaviour of the investigated mutants. Of note, GIST cell lines bearing PDGFRα mutations are not available.

## Results

### Wild-type PDGFRα only weakly activates STAT transcription factors

**Figure 1:**
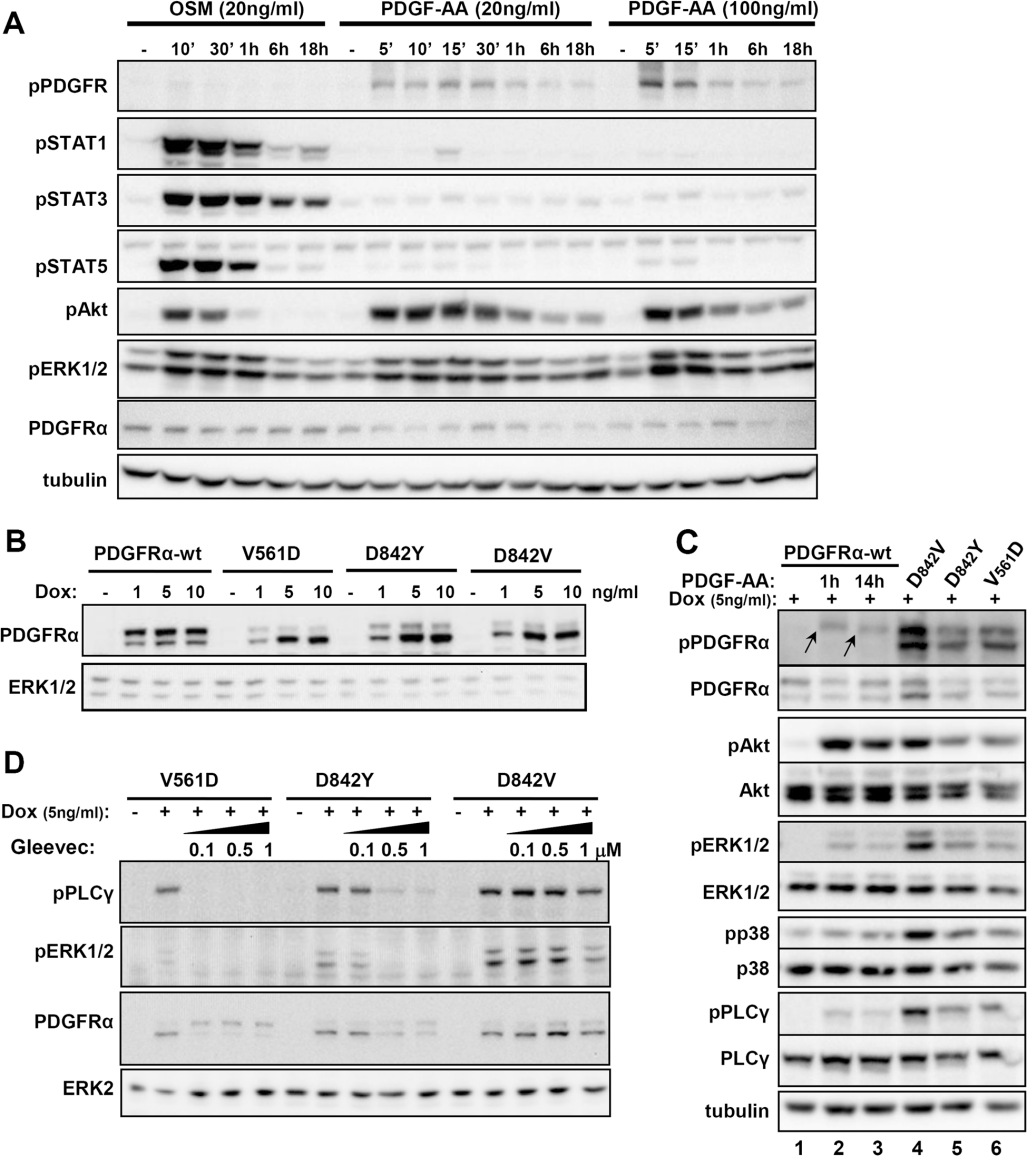
**Conventional signalling via PDGFRα wild-type and oncogenic mutant proteins. (A)** Primary NHDF (P303) cells were treated with the indicated concentrations of OSM and PDGF-AA for the times indicated and phosphorylation of PDGFR, STAT1, STAT3, STAT5, AKT and ERK was monitored by Western blot analysis. A tubulin stain is provided as control. **(B)** 293FR-PDGFRα cell lines stably expressing PDGFR-wt or mutant proteins were treated with 1, 5 or 10 ng/ml doxycycline for 20 h. Cell lysates were analysed by immunoblotting with anti-PDGFRα antibody. An ERK1/2 staining is represented as loading control **(C)** 293FR-PDGFRα stable cell lines were treated with doxycycline for 14 h and PDGFRα wild-type cells were additionally treated with PDGF-AA for the indicated times (lanes 2,3). Activation and expression of AKT, ERK1/2, p38 and PLC-γ was assessed by Western blot analysis. A tubulin stain is provided as control. **(D)** 293FR-PDGFRα cell lines treated with doxycycline for 14 h were additionally incubated with the inhibitor Gleevec for 14 h as indicated. Activation of ERK1/2 and PLCγ was assessed by Western blot analysis. An ERK2 staining is represented as loading control.

In order to evaluate the extend of signalling via the PDGFRα, we compared the signalling capacities of the Interleukin-6-type cytokine Oncostatin M (OSM) and PDGF-AA in primary human dermal fibroblasts. Figure 1A shows that PDGF-AA, which primarily signals via the PDGF receptor alpha, induces a prominent and long lasting activation of the PDGFR, AKT and ERK1/2. However, the activation of the STAT transcription factors STAT1, STAT3 and STAT5 only occurs very transiently and is extremely weak if compared to a classical cytokine signal initiated by OSM. This finding could be confirmed in two additional primary fibroblast cell lines (one of which is represented in Additional file 1: Figure S1B). When investigating the activation of the MAP-kinase p38, we observed that PDGF-AA also leads to a relatively weak and very transient phosphorylation of p38 (Additional file 1: Figure S1A and B).

### Conventional signalling initiated by oncogenic PDGFRα mutant proteins

For the inducible expression of the wild-type receptor or the different mutants, we used an isogenic system based on the 293 cell line which does not express PDGF receptors.

The cell line was equipped with a Flp recombinase target (FRT) site for the integration of the genes of interest to avoid artefacts due to random transgene incorporation. Protein expression was induced by treatment with doxycycline. Induction of receptor expression with doxycycline (Dox) was generally performed for a total of 14 h in order to allow receptor levels to stabilise. Stimulations of the wild-type receptor were also performed for 14 h to facilitate the comparison of the wild-type signals with those of the constitutively active mutants. A 1 h stimulation with PDGF-AA was usually also included for the wild-type protein to monitor the onset of signalling responses. Figure 1B shows the inducible expression (doxycycline dose–response experiment) of the wild-type receptor as well as of the oncogenic PDGFRα-V561D, −D842V and D842Y mutant proteins. Figure 1B reveals that wild-type and mutant PDGFRα proteins appear as two distinct bands in the Western blot. Moreover, the distribution of the total protein between those two fractions, which is reflected by the relative intensities of the two receptor bands, differs significantly between the wild-type and mutant proteins. Subsequent experiments were performed using 5 ng/ml doxycycline as this was the lowest concentration leading to comparable expression levels.

We first monitored conventional signalling pathways typically activated by binding of PDGF-ligands to their wild-type receptors (Figure 1C). Staining with a phosphotyrosine-specific PDGFRα antibody recognizing the activation loop tyrosine highlights the constitutive activation of the mutant proteins (on both receptor bands, Figure 1C, lanes 4–6) and confirms the stimulation-dependent activation of the wild-type receptor (on the upper receptor band only, highlighted by arrows in Figure 1C, lanes 2 and 3). It must be stressed that PDGF-AA, which generally leads to weaker signalling responses than other PDGF-family members such as PDGF-BB, led to a comparatively weak phosphorylation of the receptor itself (even at high saturating concentrations of 250 ng/ml). However, the activation of downstream signalling components was comparable to the mutant receptors. Whereas ERK1/2 and PLCγ activation was generally weaker for the wild-type receptor, AKT activation proved to be generally more efficient in the case of the wild-type protein (Figure 1C, lanes 2 and 3). The activation of the MAP kinase p38 was hard to monitor as it was relatively weak in case of the mutant proteins (Figures 1C). In our experiments we could not observe significant p38 activation via the wild-type receptor. This is not surprising as p38 activation can also be weak in primary fibroblasts (Additional file 1: Figure S1B). Consequently, as our mutant proteins showed a dramatically prolonged and also increased activation of p38 we decided to include the p38 pathway into the “unconventional signalling” for the later bioinformatic analyses. Overall, our experiments highlight a hyperphosphorylation of the receptor in the case of the constitutively active mutants (Figure 1C; lanes 4–6). This hyperactivation is part of the mutant phenotype and may also affect downstream signalling behaviour. All of the mutants activate the conventional PDGFRα signalling pathways.

Next, we studied the sensitivity of the different PDGFR mutants for treatment with the tyrosine kinase inhibitor (TKI) Gleevec (Imatinib mesylate), a drug used in the clinics for the treatment of GIST patients. For this, we monitored the dose-dependent inhibition of the downstream signalling molecules PLCγ and ERK1/2 (Figure 1D). As previously described [9], we found that the three mutants have different sensitivities for Gleevec with PDGFRα-D842V being least sensitive to the treatment and the V561D mutant being most sensitive.

### Unconventional activation of STAT factors by mutant PDGFRα proteins

**Figure 2:**
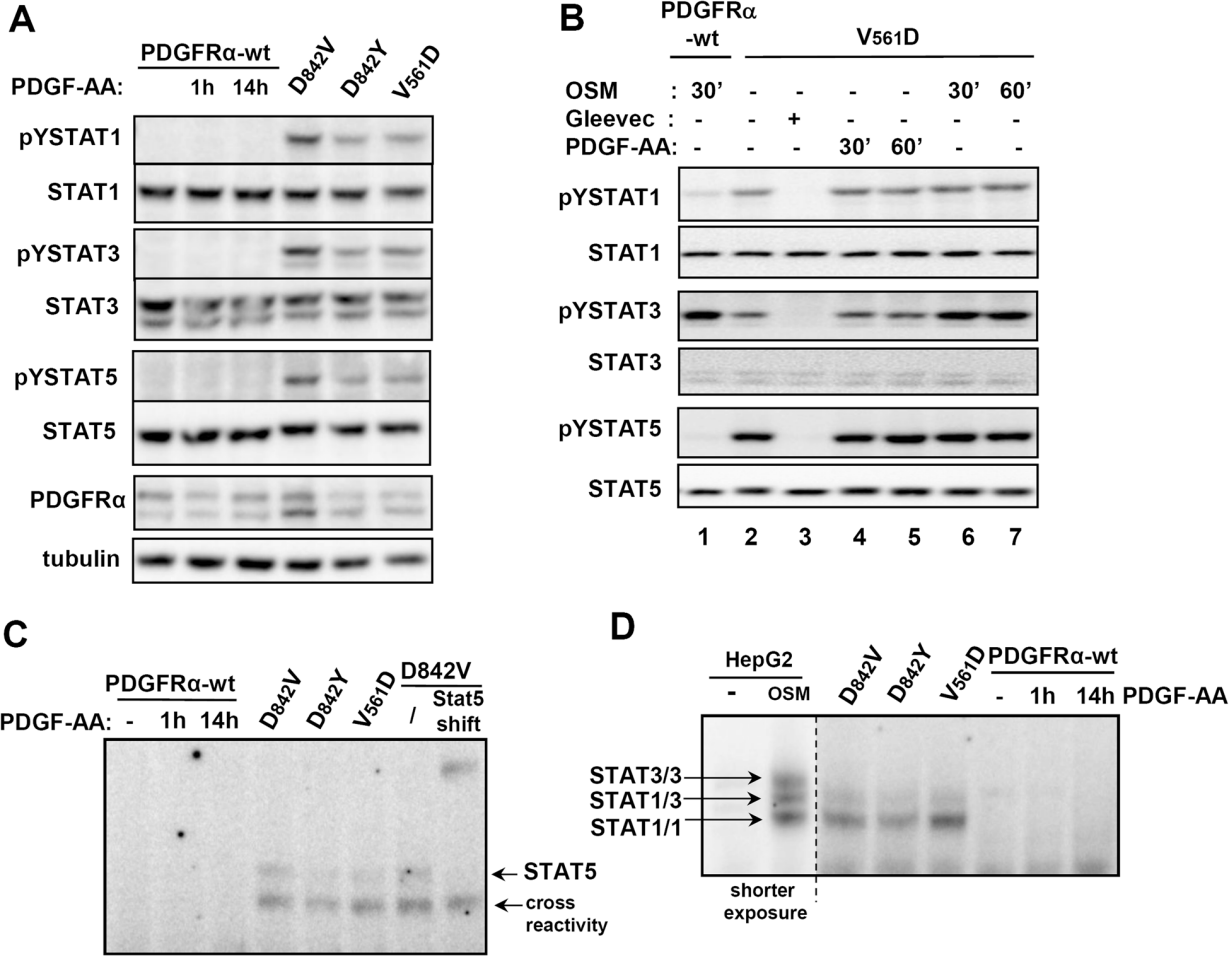
**Unconventional signalling via oncogenic PDGFRα mutant proteins. (A)** 293FR-PDGFRα stable cell lines were treated with doxycycline for 14 h and PDGFRα wild-type cells were additionally treated with PDGF-AA for the indicated times. Activation and expression of STAT1, STAT3 and STAT5 were assessed by Western blot analysis. **(B)** 293FR-PDGFRα-wt cells were stimulated with OSM (25 ng/ml) for 30 min (lane 1). Activation of STAT factors was compared with the constitutive signals detected in 293-FR-PDGFRα-V561D cells (lanes 2–7). **(C)** 293FR-PDGFRα stable cell lines were treated as described for (A). Nuclear extracts were prepared and the formation of STAT5-DNA complexes was analysed by Electrophoretic Mobility Shift Assay (EMSA) using a β-casein oligonucleotide. The identity of the STAT5/DNA complex was confirmed by super-shift experiments with a STAT5 antibody. **(D)** 293FR-PDGFRα stable cell lines were treated as described for **(B)** and the formation of STAT1/STAT1-, STAT1/STAT3- and STAT3/STAT3-DNA complexes was analysed by EMSA using an SIE oligonucleotide. As a control for the formation of the different STAT1 and STAT3 complexes, HepG2 cells were treated with OncostatinM and analysed on the same gel. The positions of the STAT/DNA complexes are indicated by arrows.

Since persistent STAT activation often occurs in cancer, we were interested whether the oncogenic activation of PDGFRα also leads to the activation of STAT1, STAT3 and STAT5 transcription factors. Figure 2A shows that the three investigated oncogenic mutants induce phosphorylation of STAT1, STAT3 and STAT5. In contrast, stimulation of the wild-type PDGFRα by PDGF-AA does not lead to a significant phosphorylation of these STAT proteins. In order to verify whether the activation of STAT factors via PDGFRα wild-type may occur transiently at very early time points, we performed short-time kinetics in PDGF-AA stimulated 293FR-PDGFRα-wt cells. Again, an activation of STAT factors was not apparent (Additional file 1: Figure S1C). This is entirely in line with our observations in primary human fibroblasts (Figure 1A and Additional file 1: Figure S1A and B). In order to compare the STAT activation via the mutated PDGFRα receptors with a cytokine-induced activation we stimulated PDGFRα-wt expressing cells with OncostatinM (OSM) and compared STAT1, STAT3 and STAT5 phosphorylation with the constitutive STAT signals in PDGFRα-V561D cells (Figure 2B). As expected, stimulation of the wild-type cells with OSM induced a strong STAT3 phosphorylation (Figure 2B, lane 1). Phosphorylation of STAT1 was also detectable but STAT5 phosphorylation elicited by OSM proved to be rather weak in 293 cells. The comparison with the V561D mutant protein (lane 2) demonstrates that STAT3 activation via the mutant protein is weaker in comparison to OSM but that STAT1 and STAT5 activation are stronger. All of the signals can be abrogated by treatment with Gleevec (lane 3) and STAT3 activation in the mutant cell line can be increased by additional stimulation with OSM (lanes 6 and 7). The activation of STAT factors via the mutant receptors is thus comparable to the intensity of cytokine-induced STAT activation.

To study if the oncogenic phosphorylation of STAT1, STAT3 and STAT5 also promotes the formation of DNA binding competent STAT complexes, we performed electrophoretic mobility shift assays. Figure 2C illustrates that all three mutants (PDGFRα-V561D, −D842V and -D842Y) induce STAT5 DNA-binding activity. The specificity of the STAT5/DNA complex was confirmed by shifting the band using a STAT5 antibody. As STAT1 and STAT3 can form STAT1/STAT1, STAT3/STAT3 homodimers as well as STAT1/STAT3 heterodimers we used a mutated SIE element known to be suitable for the detection of these three STAT species. As a positive control, we used lysates of HepG2 cells stimulated with OncostatinM (OSM). As shown in Figure 2D, the most prominent STAT species activated by the mutant receptors was the STAT1/STAT1 homodimer. A STAT1/STAT3 heterodimeric species was also detectable but a STAT3/STAT3 homodimer was not observed. This suggests that the phosphorylated STAT3 is primarily present in the form of STAT1/STAT3 heterodimers. As the formation of STAT1/STAT1 homodimers seems to be required for an efficient STAT1 response [16], our result strongly suggests that the oncogenic mutants can elicit STAT1 responses.

### Constitutive activation of oncogenic PDGFRα mutant proteins alters their glycosylation pattern

**Figure 3:**
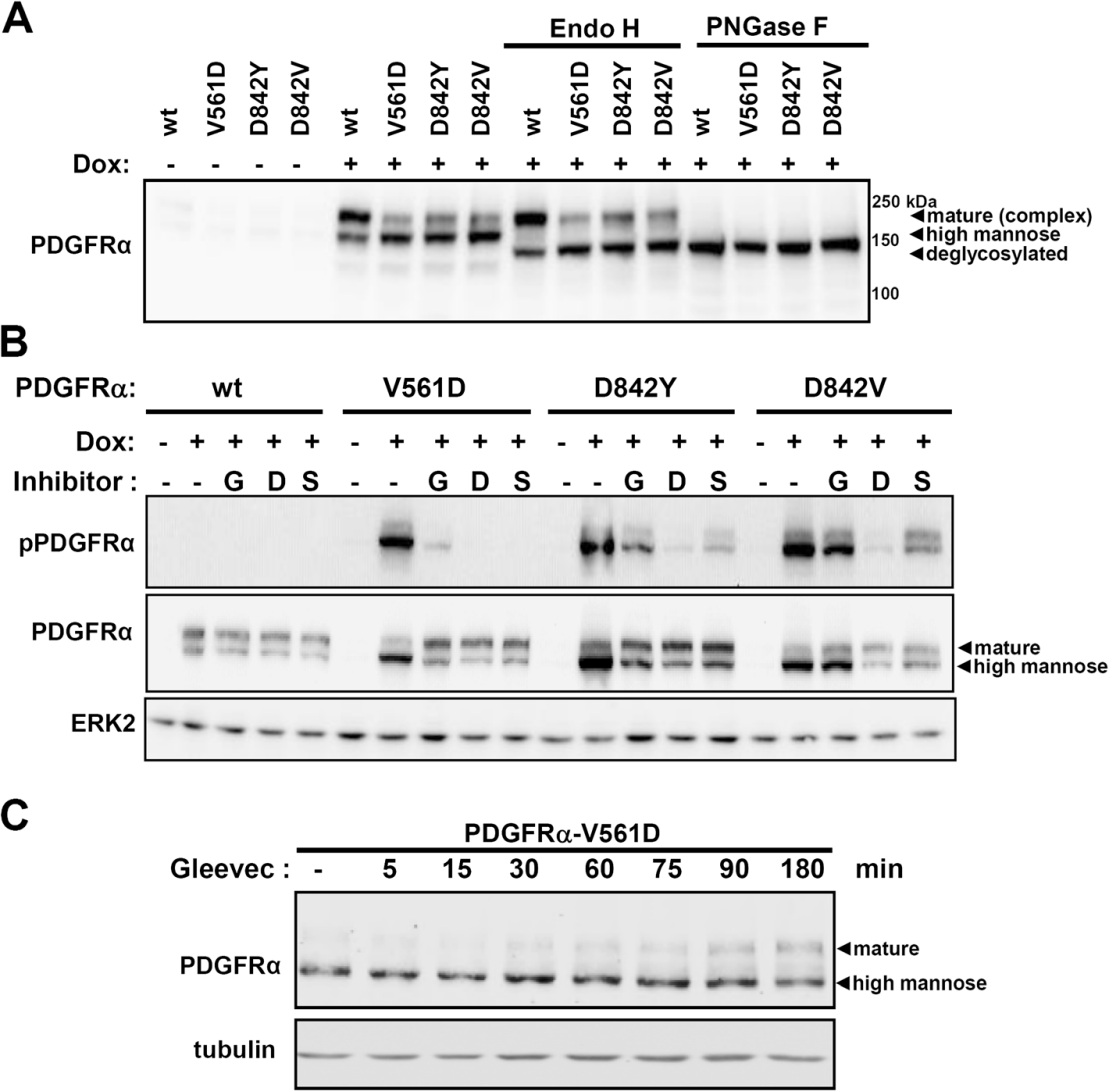
**Incomplete glycosylation of mutant PDGFRα. (A)** 293FR-PDGFRα stable cell lines were either treated with doxycycline (5 ng/ml) for 10 h or were left untreated (uninduced). Collected total cell lysates were either left untreated or were treated with Endo H or PNGase F in order to remove mannose or complex glycans from glycoproteins, respectively. The different glycosylated forms of PDGFRα were investigated by Western blot analysis using a PDGFRα antibody **(B)** 293FR-PDGFRα cell lines were treated with doxycycline for 14 h. In addition, each cell line was incubated with three different PDGFR-α inhibitors (G: Gleevec (1 μM), D: Dasatinib (1 μM), S: Sunitinib (1 μM)). The activation and presence of the different PDGFRα proteins were assessed by Western blot analysis. An ERK2 staining is represented as loading control. **(C)** 293FR-PDGFRα-V561D cells were treated with doxycycline for 14 h. The cells were subsequently incubated with Gleevec (1 μM) for the times indicated and the distribution of the glycosylated bands was analysed by Western blot using a PDGFRα antibody. As a loading control, a tubulin staining was additionally performed.

As we observed differences between the wild-type and mutant PDGFRα concerning the distribution of the total protein (reflected by the relative intensities of the two receptor bands; Figure 1B) we investigated the glycosylation pattern of the receptor for the mutant and wild-type proteins. For this, we treated the cellular lysates with the glycosidases EndoH or PNGaseF in order to eliminate high mannose glycosylation or total glycosylation, respectively (Figure 3A). The observed mobility shift following the enzymatic treatment demonstrates that the upper PDGFRα band constitutes the complex glycosylated form and thus corresponds to the mature form of the receptor whereas the lower of the two bands represents a high mannose form.

In order to clarify whether the observed band pattern of the PDGFRα protein is related to its constitutive activation, we treated the constitutively active, mutated receptors with three different TKIs: Gleevec (G), Dasatinib (D) and Sunitinib (S) (Figure 3B). The latter two were included to additionally inhibit the PDGFRα-D842V mutant which is insensitive to clinically relevant Gleevec concentrations. Treatment with the inhibitor occurred simultaneously to the induction of the receptor expression with doxycycline in order to prevent any activation of the receptor. Effective inhibition was monitored by assessing the phosphorylation status of the activation loop tyrosine Tyr849 of the receptor (pPDGFRα). As shown in Figure 3B (as well as in Figure 1D, lower panel), the high mannose form of the receptor decreases upon inhibition of the mutated receptors and the effect is accompanied by a relative increase of the complex glycosylated form.

To investigate whether short term inhibition of an activated receptor also affects the relative intensities of the receptor bands we induced the expression of the PDGFRα-V561D mutant in the absence of Gleevec. Subsequently, we treated the cells with Gleevec for 5 min to 180 min (Figure 3C). We detected a gradual increase of the complex glycosylated form of the receptor over the investigated time frame which was visible about 30 minutes after Gleevec treatment. This experiment also demonstrates that the aberrant glycosylation pattern of the constitutively active receptor can be reversed by treatment with TKIs. Taken together, these results show that a large pool of the oncogenic mutant receptors is present as high mannose form and that the over-representation of this form is linked to their constitutive activity.

### Constitutively active mutant PDGFRα proteins show retention in the ER/Golgi compartment

**Figure 4:**
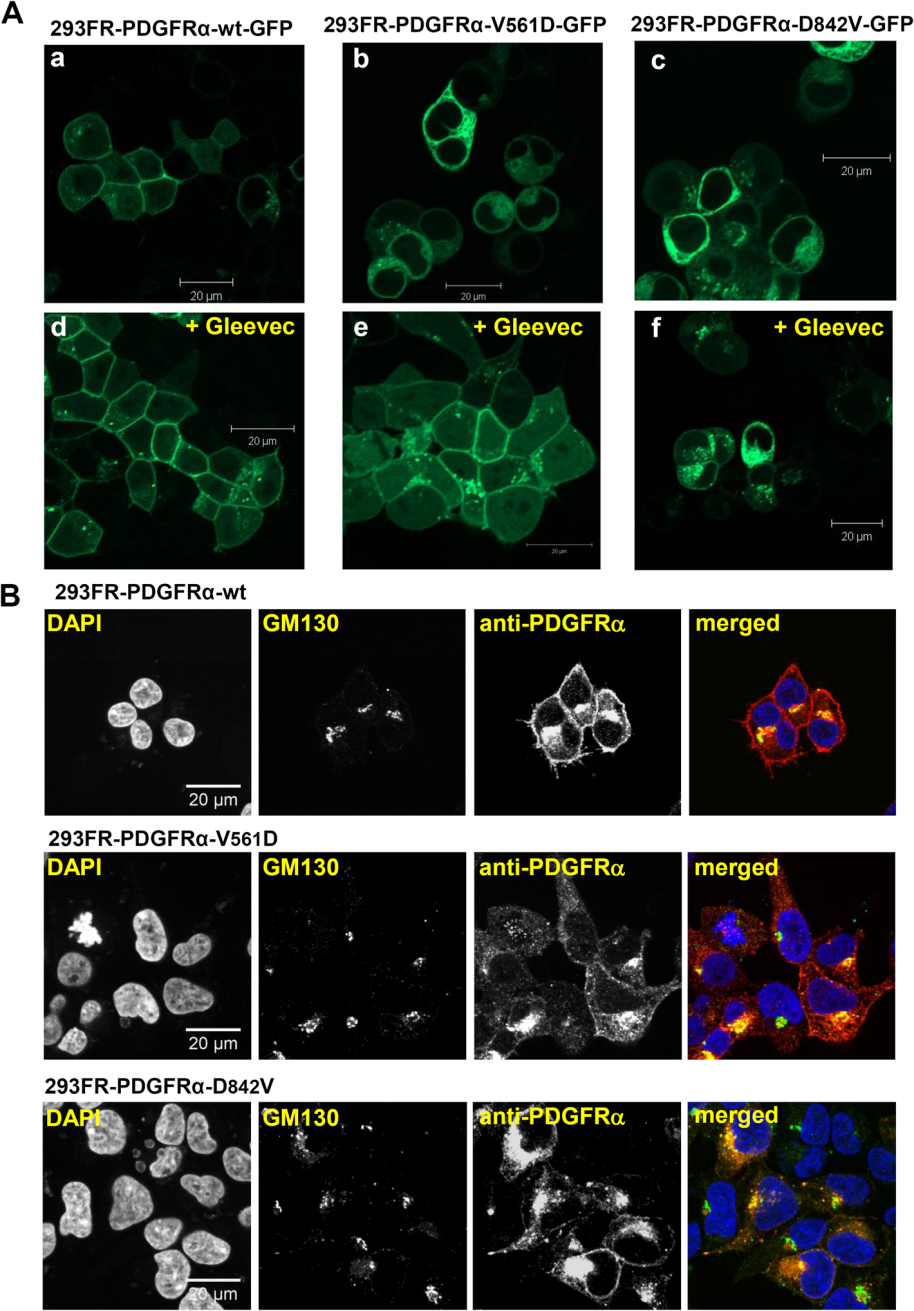
**Activity-dependent mislocalisation of mutant PDGFRα. (A)** 293FR-PDGFRα-GFP stable cell lines were treated with doxycycline for 14 h and subjected to confocal live cell microscopy. For figures d-f, the cells were additionally treated with 1 μM Gleevec for 14 h. **(B)** 293FR-PDGFRα stable cell lines were treated with doxycycline for 14 h, fixed, permeabilized and stained with DAPI (nuclear marker), GM130 (Golgi marker) and anti-PDGFRα antibody as described in the Methods section. The merged image represents PDGFRα proteins in red, the Golgi compartment in green, the nucleus in blue and PDGFRα/Golgi colocalisation in yellow.

**Figure 5:**
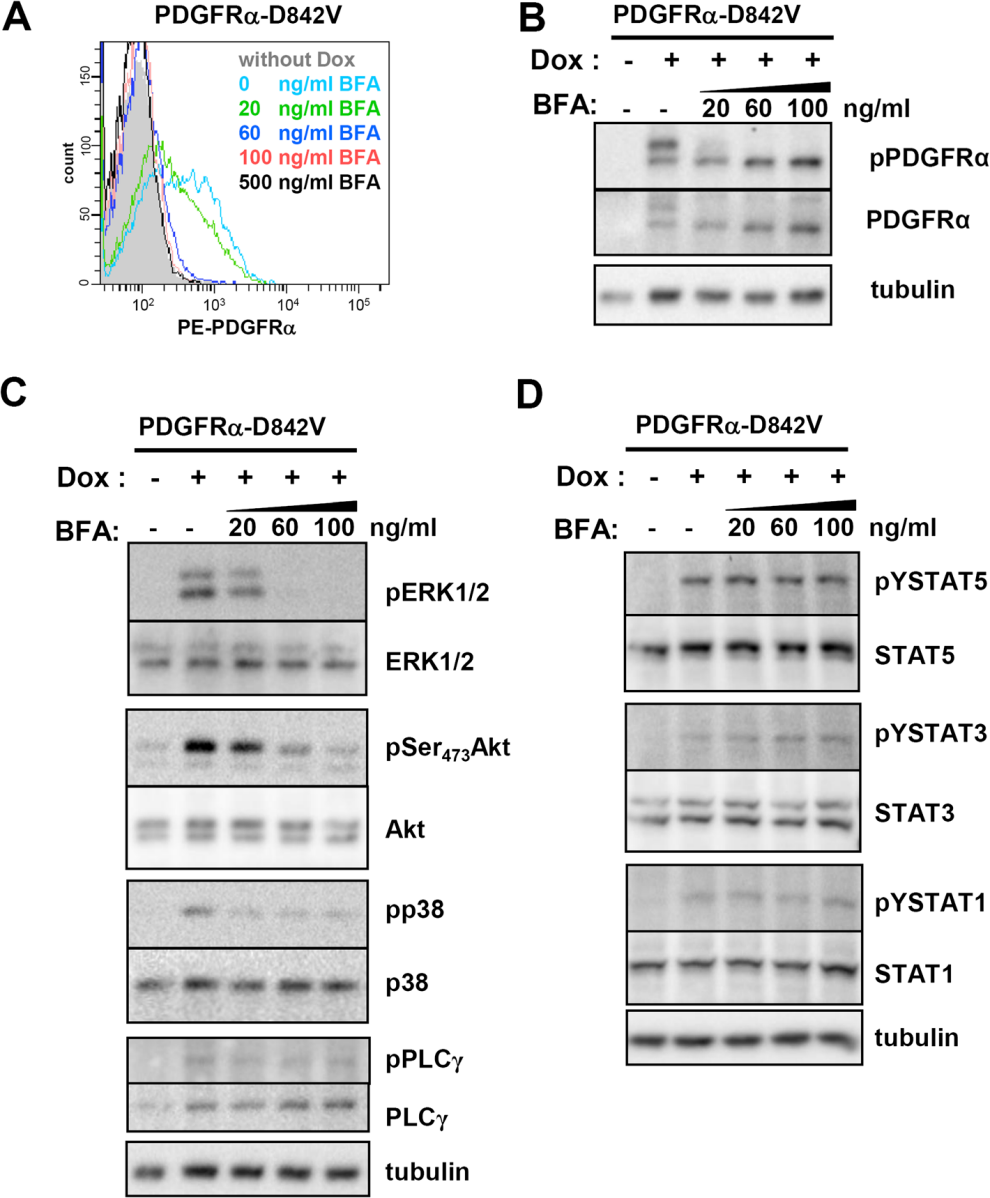
**Differential signalling of mutant PDGFRα from the ER and plasma membrane. (A)** Flow cytometric analysis of PDGFRα surface expression in 293FR-PDGFRα-D842V cells treated with doxycycline for 4 h in the presence of the indicated amount of BrefeldinA (BFA). **(B)** 293FR-PDGFRα-D842V cells were treated as described in **(A)**. After cell lysis, the different glycosylated forms of the receptor were visualized using antibodies recognizing pPDGFRα or PDGFRα. **(C)** Cells were treated as described above and the effects of Golgi-destruction on conventional PDGFRα signalling were assessed by Western blot analysis using the corresponding antibodies. **(D)** The effects of Golgi-destruction on unconventional PDGFRα signalling via STAT factors were assessed by Western blot analysis using the corresponding antibodies.

Our previous experiments demonstrated that inhibition of the kinase activity induced changes in the glycosylation pattern of the oncogenic mutants. Next, we studied the localization of the oncogenic mutants in comparison to the wild-type PDGFRα. Therefore, we generated stable, inducible cell lines expressing GFP-tagged PDGFRα-wt, −V561D, or -D842V receptors and monitored their localization in living cells using laser scanning microscopy. In order to study whether the distribution of the protein is influenced by the constitutive activation of the mutant receptors, we monitored cells which were either treated with Gleevec or were left untreated. Figure 4A a-c show that the localization of wild-type receptors differs considerably from the oncogenic mutants: Without inhibitor treatment, the wild-type receptor displays a clear membrane localization in contrast to the constitutively active V561D and D842V proteins, which in turn show strong signals from intracellular compartments, mainly the ER/Golgi compartment. We took advantage of the different sensitivities of the two oncogenic PDGFRα mutants towards the inhibitor Gleevec and tested the activity-driven redistribution of the mutant proteins. Reduced intracellular and increased plasma membrane-localisation of the Gleevec-sensitive PDGFRα-V561D can be observed upon inhibition of its constitutive activity (Figure 4A, b vs e). Importantly, the Gleevec-insensitive D842V mutant remains in the cytosolic compartment (Figure 4A, f), clarifying that the increased intracellular signal depends on the constitutive activation of the mutant proteins. Due to the intensive intracellular retention of the mutant proteins, a simultaneous surface localization of sub fractions of total protein is difficult to evaluate using confocal microscopy. However a membrane localized pool of mutant receptors can be detected using FACS analysis (Figure 5A), showing that a fraction of the mutant receptors reaches the plasma membrane. In order to confirm the retention in the ER/Golgi compartment we next performed a co-staining of the PDGFRα and the Golgi in 293FR cells expressing PDGFRα wild-type and mutant proteins (Figure 4B). As expected, the wild-type PDGFRα (upper panels) displays a prominent plasma membrane localisation. However, the mutant proteins (middle and lower panels) show a clear shift from a plasma membrane to intracellular localisation and display a pronounced co-localisation with the Golgi marker GM130. These results further confirm the mislocalisation of the mutant PDGFRα proteins.

### Differential signalling of intracellular and plasma membrane pools of oncogenic PDGFRα mutant proteins

To evaluate whether the observed non-conventional activation of STAT factors by the oncogenic mutants is linked to their mislocalisation, we investigated the signalling capacities of PDGFRα-D842V in the presence of Brefeldin-A (BFA). BFA interferes with the transport of proteins from the endoplasmic reticulum to the Golgi compartment. Importantly, BFA treatment was performed simultaneously with induction of receptor expression using doxycycline so that the induced receptors are incapable of reaching the plasma membrane during the experiment. This is confirmed by our FACS analysis (Figure 5A) and allows excluding that the observed signalling occurs after receptor internalisation following receptor surface expression. We performed a dose–response experiment with BFA and monitored cell surface expression of the receptor using FACS analysis. Figure 5A demonstrates that plasma membrane localisation of PDGFRα-D842V is prevented at a BFA-concentration of 60 ng/ml. Detection of the PDGFRα-D842V protein from the corresponding lysates shows that abrogated cell surface expression is paralleled by the disappearance of the complex glycosylated form of the mutant protein (Figure 5B). When monitoring the conventional PDGFR signalling (Figure 5C), we observe that the loss of cell surface expression also strongly impairs the activation of ERK1/2, AKT and p38. Most interestingly, the non-conventional signalling that we identified, namely the activation of the STAT factors STAT1, STAT3 and STAT5, was not altered by pre-treatment with BFA (Figure 5D). This shows that STAT factor activation via the oncogenic mutants does not require plasma membrane localisation and originates from the intracellular pools of the mutant proteins. Phosphorylation of PLCγ was apparently also not dependent on plasma membrane localisation (Figure 5C).

### Different PDGFRα mutants display virtually identical biological responses

**Figure 6:**
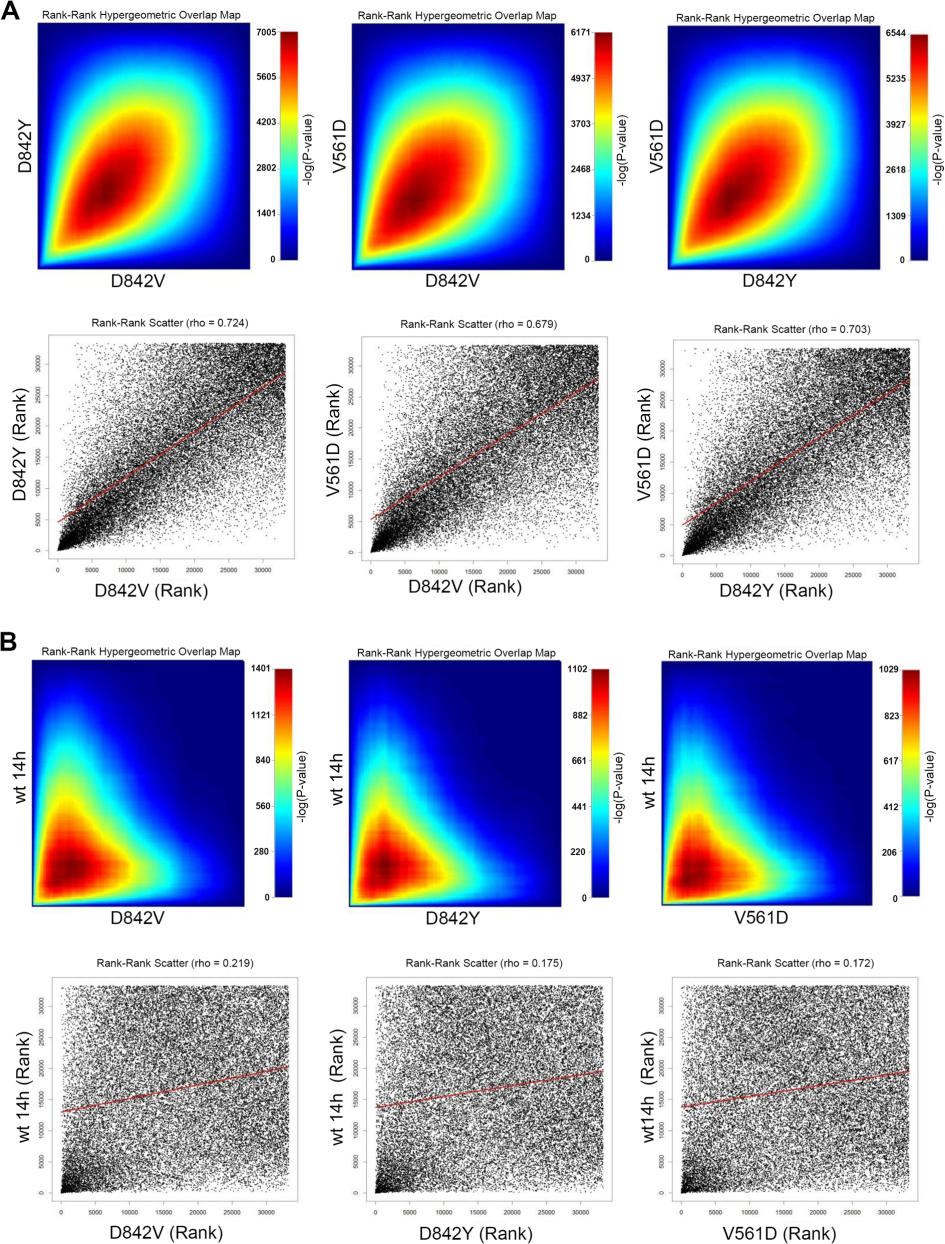
**Different mutant PDGFRα proteins show highly similar biologic responses. (A)** RRHO heatmaps (upper row) and rank-rank scatter plots (lower row) for the comparison of the different oncogenic PDGFRα proteins. The representation is based on our microarray experiments and does not apply any cut-offs as the entire gene set is used in the analysis. For both lists, the genes were ranked according to the ANOVA p-values attributed to the differentially expressed genes (using non-stimulated PDGFRα-wt as control). The top differentially expressed genes are thus located at the lower left corner of the graph. For the heat maps, the range of -log10-transformed hypergeometric P-values are indicated in the colour scale bar. High intensity signals (red) indicate the highest overlap in the lists above the current sliding rank threshold (between the rank 1 for both lists at the bottom left corner and the coloured point on the map). **(B)** RRHO heatmaps (upper row) and rank-rank scatter plots (lower rows) for the comparison of stimulated PDGFRα response and the different oncogenic PDGFRα mutants.

Having established that the oncogenic PDGFRα mutants displayed an identical signalling pattern we next compared their gene expression profile. Therefore we monitored the transcriptomic profiles of stimulated PDGFRα-wt (14 h PDGF-AA) as well as of the three mutant proteins. Avoiding arbitrary cut-off setting, we first performed a rank-rank hypergeometric overlap (RRHO) analysis [17] in order to compare the overall gene expression signature of the mutant proteins. This analysis identifies the statistically significant overlap while stepping through two lists of genes which have been ranked by their differential expression (in our case, the step-up P-value related to their differential expression in regard to non-stimulated PDGFRα-wt expressing cells). The significance of the overlap of the two lists above this sliding rank threshold is represented as a heatmap [17]. This method thus indicates the similarity between two gene lists by comparing the ranking of the genes in the corresponding lists (more details on this technique are provided in the materials and method section 4.8.). The RRHO analysis shows that the gene expression signatures of the three mutant proteins are highly similar (Figure 6A, RRHO heat maps). Figure 6A also shows the corresponding rank-rank scatter plot in which all the genes are represented as individual dots and in which high ranking regulated genes are regrouped in the lower left corner. The high intensity signal along the diagonal axis in the lower left corner of all three comparisons highlights that the gene lists contain a very high number of common high ranking genes. This is absolutely in line with our observation that the three mutants share a common signalling pattern which is independent of the location of the mutation within the receptor chain (Figures 1 and 2). In contrast, the comparison of the transcriptomic signatures of the mutants with the stimulated wild-type receptor displays clear differences in the expression patterns (Figure 6B). The rank-rank scatter plot shows a much more random distribution and the density in the lower left corner does not follow the diagonal axis to the same extent. This highlights that there are significant differences in the ranking of the top genes between the wild-type protein and the three mutant receptors. Overall, the RRHO analysis thus shows that the observed divergent signalling patterns of mutant and wild-type proteins also translate into a divergent response.

### Comparison of the signatures of wild-type and mutant PDGFRα proteins reveals differences in the biological responses

**Figure 7:**
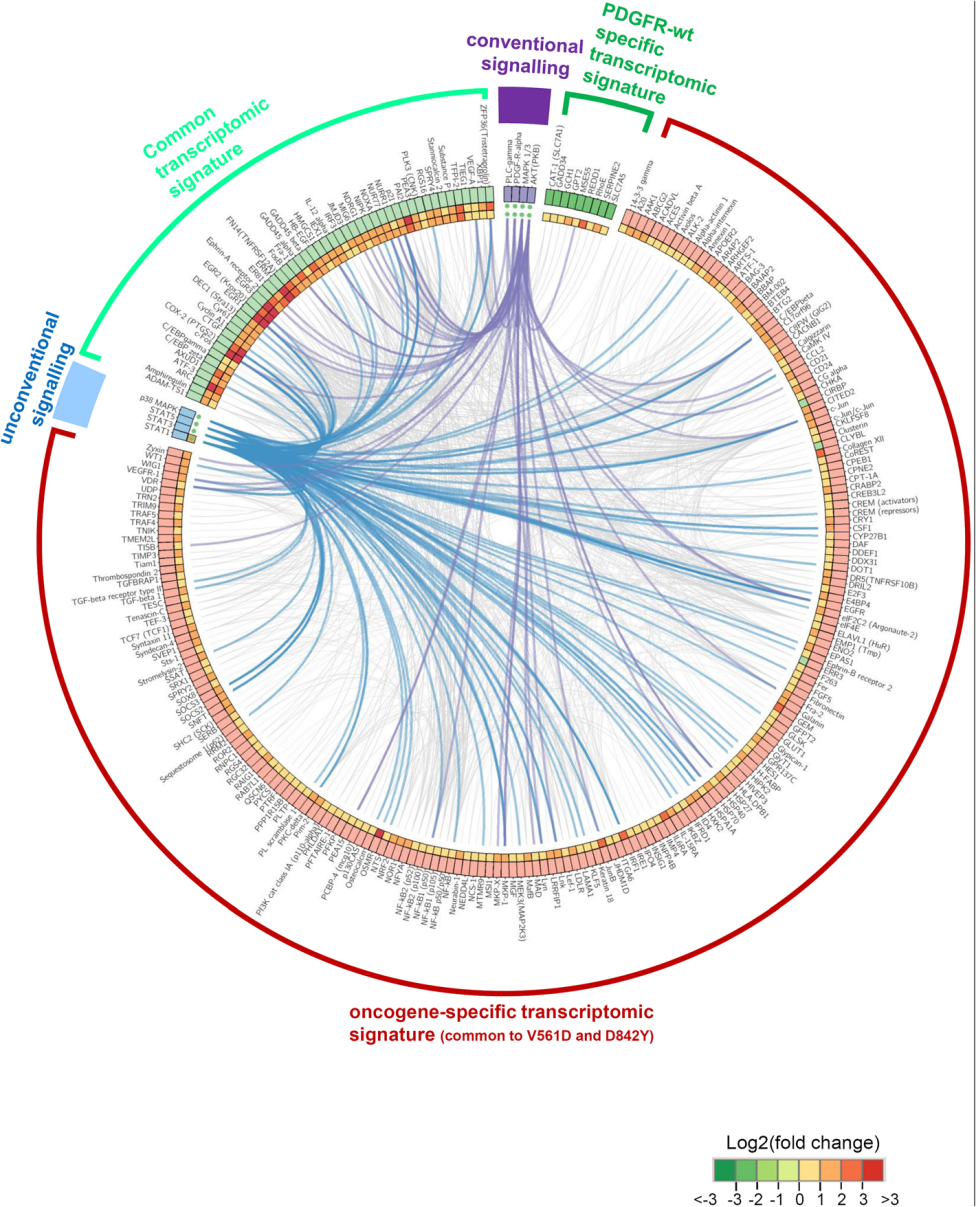
**Common and divergent biologic responses initiated by wild-type and mutant PDGFRα.** Circos plot [68] representing the generated signalling/transcriptomic gene regulatory networks from the microarray analyses. The figure shows an overlay of the *global PDGFRα-mutant regulatory network* (common signature of the V561D and D842Y mutants) and the stimulated *PDGFRα-wt regulatory network* (see materials and methods section for details on network generation). Only SDEGs with a step-up FDR less than 0.05 and absolute fold change greater than 40% (in comparison to non-stimulated PDGFRα-wt control cells) are represented. The SDEGs were divided into three groups: 1) the common regulated genes between the oncogenic situation and the PDGF-AA stimulated wild-type protein (highlighted in light green), 2) SDEGs which are exclusively regulated under the oncogenic situation (red), 3) SDEGs which are only regulated for the PDGF-AA-stimulated (14 h) wild-type receptor (dark green). The average log2 transformed fold change between the corresponding situations and control is represented as a heat map in the two circles (outer heat map circle: merged V561D/D842Y; inner heat map circle: PDGF-AA stimulated receptor). The observed signalling characteristics are represented as conventional (violet) and unconventional (blue) signalling. The activation of these signalling components by the mutant or the wild-type receptors is indicated by green dots. The interactions between the molecules in the networks were visualized as violet (conventional signalling to transcriptomic responses), blue (unconventional signalling to transcriptomic responses) or grey (transcriptomic to transcriptomic) connections.

In order to compare the oncogene-specific transcriptional responses to those of the wild-type receptor, we investigated how the differential and common responses are linked to the observed signalling behaviour. For this we generated several merged signalling/transcriptomics regulatory networks using the MetaCore® platform (details on the network generation are provided in the materials and methods section, 4.9.). First, we compared the common transcriptomic signature of the two mutants V561D and D842Y (*global PDGFRα-mutant regulatory network*) to the signature of the PDGF-AA stimulated wild-type receptor (*PDGFRα-wt regulatory network*). The circos plot shown in Figure 7 integrates the transcriptomic signatures (common to wild-type and mutant proteins (light green), oncogene-specific (red) and wild-type specific (dark green)) with the identified activated signalling components (conventional signalling (violet); unconventional signalling (blue)). Only differential regulated genes with an FDR below 0.05 and an absolute fold change exceeding 40% (in comparison to non-stimulated PDGFRα-wt control cells) are indicated in Figure 7. The figure illustrates that signalling via STAT factors is an integral part of the oncogenic network generated based on the MetaCore knowledge database and that it is strongly linked to the oncogene-specific transcriptomic signature. Of note, in Figure 7 the unconventional signalling pathways are not exclusively linked to the oncogenic response. Such effects are due to the fact that there are many reported overlaps between the signatures of the different pathways. As the interactions are based on a knowledge database, the figure does also not consider potential cross-talk mechanisms between the pathways that may exist in our specific system. However, the figure allows extracting the most prominently regulated genes for the oncogenic or wild-type situation based on the provided heat map. Furthermore, it also allows identifying bona fide STAT target genes among the oncogene-specific responses such as IRF-1, SOCS2, SOCS3, pim2. Connections between genes that are part of the transcriptomic response are indicated in grey. Of note, some of the highly connected genes such as CEPBbeta and c-Jun are also linked to the STAT factors.

Furthermore, we constructed a strongly connected components (SCC) core network based on the common signature of the V561D and D842Y mutant proteins (*global PDGFRα-mutant regulatory network*). The state of any node in an SCC possesses the potential to directly or in-directly affect the state of any other node. This structure, which potentially determines the stability of the corresponding regulatory network is visualised in the circos plot in Additional file 1: Figure S2. Again, the STAT factors appear as central and highly connected hubs in this merged signalling/transcriptomic network. In addition, we also constructed a strongly connected components network based on the *oncogene-specific PDGFRα network* that just considered oncogene-specific responses and oncogene-specific signalling components. Once more, the STATs appear as important components if only the oncogene-specific signature is considered (Additional file 1: Figure S3).

**Figure 8:**
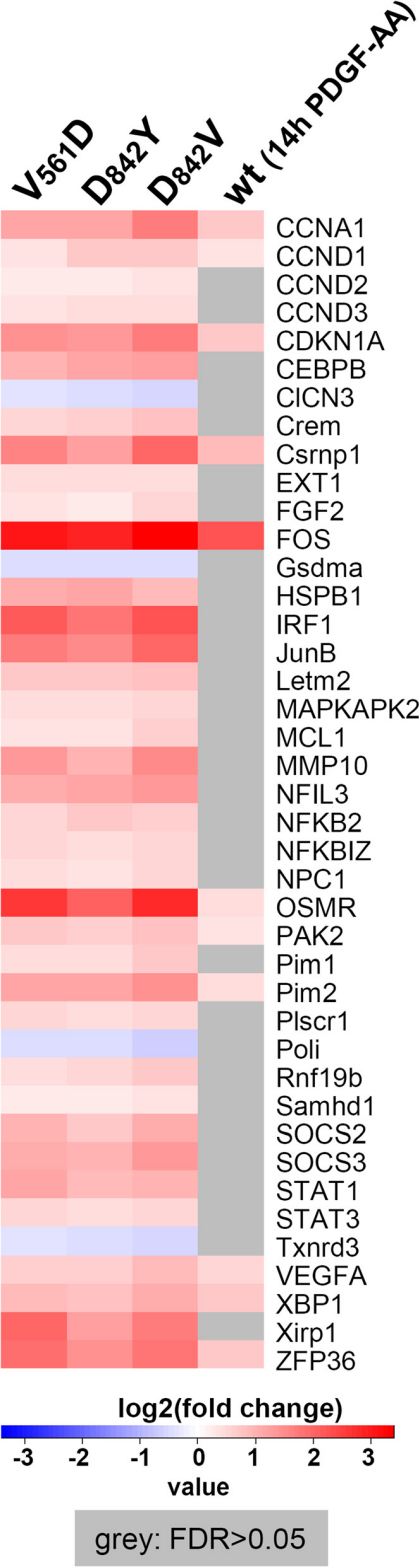
**Mutant receptors show increased expression of known STAT target genes.** Heat map showing the log2-transformed fold change of selected known STAT target genes in our microarray experiments for the PDGF-AA stimulated PDGFRα-wt and the oncogenic mutants. Conditions for which the genes showed no statistically significant alteration in expression level were coloured in grey (step-up FDR > 0.05).

Finally, we checked for the induction of a selection of reported STAT1/3/5 target genes [13,18-21] in the mutant and wild-type PDGFRα (14 h PDGF-AA) transcriptomic responses. Figure 8 highlights that the oncogenic mutants lead to the induction or increased expression of many STAT target genes whereas stimulation of the wild-type receptor fails to do so in most cases. Some genes (e.g. c-FOS, OSMR) are known to be also regulated via other signals such as ERK1/2 mediated signalling so that their regulation via PDGFRα-wt does not interfere with the observed signalling pattern. Of note, these signals tend to be increased by the additional activation of STAT factors by the oncogenic mutants (Figure 8).

To sum it up, we can show that the observed phosphorylation of STAT1, STAT3 and STAT5 transcription factors also clearly conducts to a transcriptomic response.

## Discussion

Upon expression of wild-type and mutant receptors we observed that the glycosylation patterns of the expressed proteins differ and that the oncogenic mutants show an increase in the high mannose form of the receptor compared to the wild-type protein (Figures 1B and 3A). We can clearly relate the accumulation of the high mannose form of the receptor to the constitutive activity of the oncogenic mutants. Furthermore, inhibition of the constitutive activity with inhibitors used for GIST treatment in the clinics restores the glycosylation pattern observed for the wild-type receptor (Figures 1D, 3B and C). Importantly, using live cell confocal microscopy, we can demonstrate that the increase of the high mannose form of the mutated receptors is paralleled by shift in localisation of these receptors, a phenomenon which is also dependent on the constitutive activity of the mutant proteins (Figure 4). Further studies will be needed to investigate the intracellular localisation and trafficking of the mutant proteins in more detail.

For the oncogenic Kit-D816V mutant identified in patients with systemic mastocytosis and corresponding to the GIST PDGFRα-D842V mutant, it was previously reported that its localisation to the ER/Golgi compartment is sufficient to transform cells to cytokine independence and to induce diseases in mice [22]. Similarly, the investigation of oncogenic KIT mutations occurring in GIST has demonstrated that KIT cell surface expression is not required for activation and oncogenic signal transduction and that its mislocalisation is linked to its activity [23,24]. Although mislocalisation of oncogenic Kit mutants has been subject to investigation, data on the localisation of PDGFRα mutant protein and possible implications of a mislocalisation for cellular signalling is sparse. In the context of a PDGFRα mutant occurring in glioblastoma, aberrant intracellular localisation was detected [25]. This constitutively active mutant harbouring a deletion in the extracellular part of the receptor also displayed a different glycosylation pattern compared to wild-type receptor and its mislocalisation was associated with increased oncogenic potential. Our study on GIST-derived PDGFRα mutants suggests that mislocalisation and altered signalling are general characteristics of constitutively active PDGFRα proteins. The observed hyperphosphorylation of the mutant proteins may contribute to the skewed signalling characteristics. Detailed investigations on the contribution of the hyperphosphorylation are hampered by the fact that activity (and thus phosphorylation) also affects the localisation of the mutant proteins (Figure 4A b vs. e). Together with the previously discussed activity-related mislocalisation observed for oncogenic Kit mutants the present study completes the picture that aberrant intracellular signalling is a general characteristic of GISTs that harbour mutations in receptor tyrosine kinases (KIT or PDGFRα). Activity-related abnormal glycosylation and mislocalisation has furthermore been reported for the receptor tyrosine kinases Flt3 and CSFR1 [26,27]. Undoubtedly, the activity-related receptor mislocalisation and the associated modified signalling characteristics represent a general phenomenon and influence the oncogenic potential of mutant RTKs. Our finding that the mislocalisation of GIST-associated PDGFRα mutant proteins can be prevented (Figure 3B) and reversed (Figure 3C) by the treatment with TKIs could be of importance for therapy. For example, immunotherapy approaches using monoclonal antibodies have shown their value for gastrointestinal malignancies [28]. Very recently, anti-KIT designer T-cells transduced with a chimeric immune receptor have proven their efficacy in targeting GIST cells that contain KIT mutations [29]. For potential approaches targeting mutated PDGFRα, its efficient expression at the cell surface would be paramount. Our data indicate that simultaneous treatment with TKIs would greatly enhance receptor surface expression and thus its targeting by monoclonal antibodies.

In view of potential therapies, understanding the signalling events and associated molecular mechanisms downstream of oncogenic RTKs and particularly treatment-refractory (TKI-resistant) mutants is of prime importance. Especially the comparison to the signals initiated by the wild-type receptors can help identifying new and potentially important players contributing to the oncogenic potential of mutant kinases. Analysing the signalling capacities of the wild-type receptor we confirmed the previously described activation of the PI3K/AKT, ERK1/2 and PLCγ signalling pathways (Figure 1C) [3,4,30]. Most importantly and in stark contrast to the wild-type receptor, the oncogenic mutants were also responsible for a strong and constitutive activation the STAT transcription factors STAT1, STAT3 and STAT5 (Figure 2). A comparative analysis of the transcriptomic profiles of the different investigated mutants (Figure 6A) reveals that the signature is extremely similar and that the location of mutations within the receptor chain does not per se influence the downstream response. Except for the known different sensitivity to TKI inhibitors, our data suggest that downstream signalling and transcriptomic events are indiscernible for the investigated mutants and that they are thus not exploitable in regard to a patient-specific treatment. Supporting our findings it can be noted that differential prognostic outcomes have not yet been reported for GIST patients harbouring different PDGFRα mutations (contrary to patients with KIT mutations) [31]. Comparison of the mutant signatures with the wild-type signature (Figure 6B) clearly shows different transcriptomic pattern, which is in line with the observed differential signalling. The comparison of the common signature of two oncogenic mutants (V561D and D842Y) with the signature of the stimulated wild-type protein (Figure 7 and Additional file 1: Figure S2 and S3) illustrates that the identified unconventional signalling components (and in particular the STAT factors) are strongly associated to the oncogenic response (composed of oncogene-specific and common transcriptional signatures). A transient phosphorylation of STAT factors has previously been reported to occur in the context of the wild-type PDGFRs including the regulation of some STAT target genes [32-37]. Our study does not necessarily contradict these data. We believe that STAT phosphorylation may be detected after stimulation of cells with the growth factors of the PDGF family in some cells but this activation is usually very transient and weak. As we observed for IL-6 type cytokine signalling that transient (although quite strong) phosphorylation of STAT1 does often not lead to a STAT1 response [16,38], we think that at least some of the reported phosphorylation of STAT factors may not lead to STAT dependent cellular responses. In the case of the mutant PDGFRα proteins, we observe a strong and long lasting activation of STAT1, STAT3 and STAT5, which will undoubtedly have a much more prominent effect on the cells and/or the tumour microenvironment. A recent paper by Velghe at al. supports our findings as the authors compare STAT5 activation via PDGF-BB to STAT phosphorylation initiated by stimulation with interleukin-3 [39]. The authors show that STAT5 activation does not increase upon PDGF-BB stimulation. In addition they demonstrate a strong constitutive phosphorylation of STAT5 induced by the PDGFRα-D^842^V mutant.

STAT activation by other oncogenic PDGFRα or PDGFRβ mutant proteins has already been described by several groups [40-42]. In most cases the exact activation mechanism is still unknown and it is very likely that the activation mechanism will vary between the two receptor forms and will probably also depend on the specific nature of the mutation.

**Figure 9:**
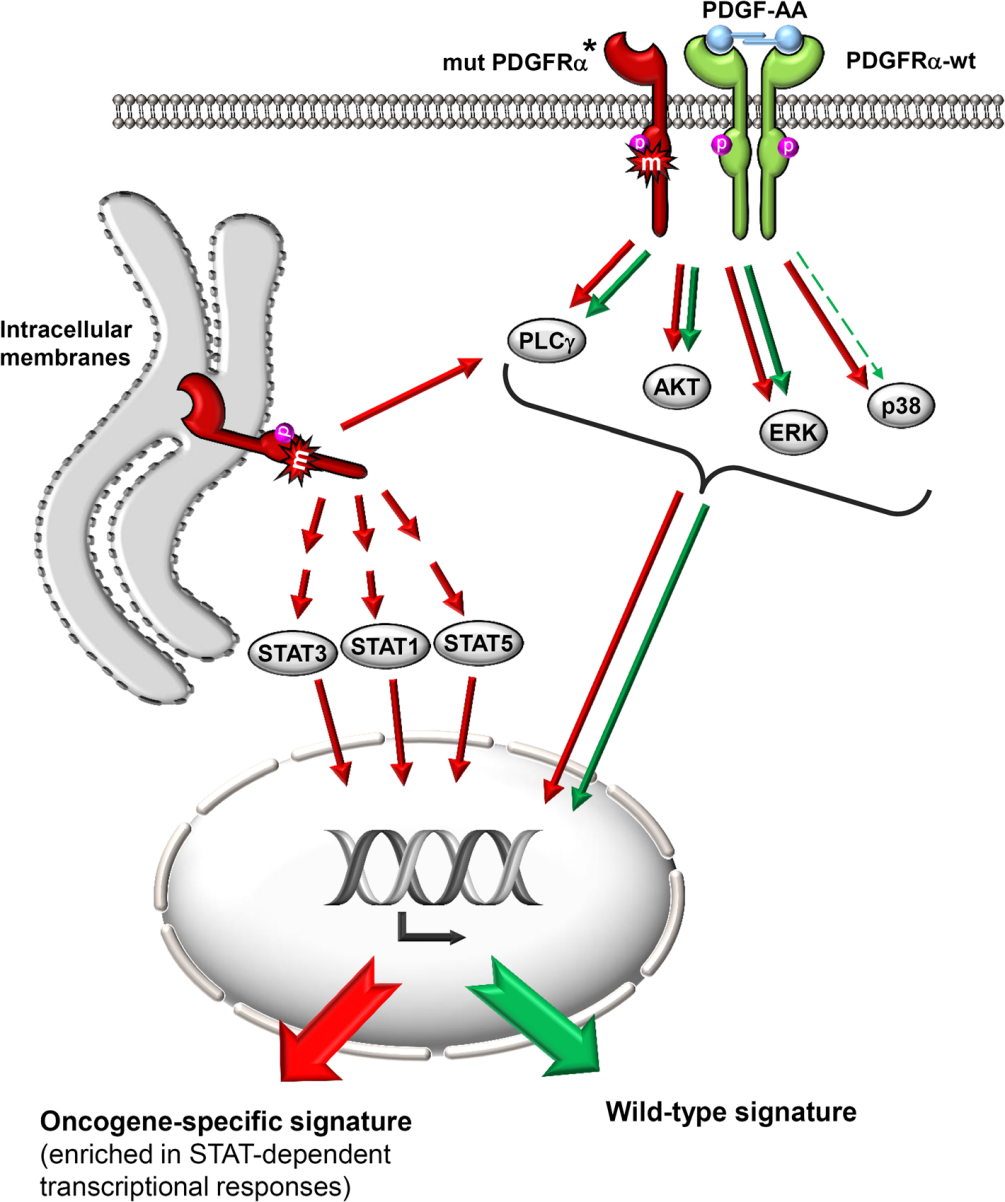
**Model recapitulating the localisation-dependent divergent signalling capacities of wild-type PDGFRα and the different pools of mutant PDGFRα proteins.** *It is currently not clear whether mutant PDGFRα proteins exist in a monomeric or dimeric state.

Aberrant and constitutive activation of STAT factors is known to be very relevant for tumourigenesis [13,14]. For example, abnormal activation of STAT5 is recognized to be important for myeloid transformation [43,44], and was also suggested to play a primordial role in leukemic transformation initiated by Flt3-ITD, an Flt3 mutant that is retained in the ER [45]. Choudhary et al. demonstrated that Flt3 signalling depends on the cellular localisation of the constitutively active mutants. To our knowledge, our findings provide the first evidence for compartment-specific signalling characteristics of mutant PDGFRα proteins. We find the mutants to be present in two major pools, one plasma membrane associated pool and one pool that signals from the ER/Golgi compartment. By abrogating protein transport from the ER to the Golgi, we demonstrate that most conventional PDGFRα signals, namely the PI3K/Akt and ERK1/2 pathways originate from the plasma membrane associated pool of the mutant kinase (Figure 9). Most importantly, we find the ER-associated fraction to mediate the observed activation of STAT1, STAT3 and STAT5. The fact that we do not observe a reduction of these signals upon disruption of the ER to Golgi transport and that the stimulated wild-type receptor does not show STAT factor activation strongly suggests that the constitutive STAT activation occurs from the intracellular, membrane-associated pool of the kinase. A contribution of kinase hyperphosphorylation can also be envisaged. Schmidt-Arras *et al.* proposed that ER associated phosphatases such as PTP1B contribute to the different signalling characteristics of the Flt3-ITD mutant receptor [26]. Interestingly, a pronounced activation of STAT1, STAT3 and STAT5 originating from ER-associated Flt3 mutants has also been reported [26,45]. Similar reports exist for c-Kit and other receptor tyrosine kinases [46,47]. However, the mechanistic details of STAT factor recruitment and direct or indirect activation via oncogenic RTKs are not understood and need to be subject to further investigations. Furthermore, the activation of several STAT factors via oncogenic mutants of Flt3, Kit or PDGFRα raises a number of important questions. First, the biological relevance in the different systems is not yet established. Although the reported importance of STAT5 activation for leukemic transformation via a Flt3 mutant is comprehensible in the myeloid system, its implications for GIST are not yet clear. However, STAT5 is also known to contribute to malignant transformation in solid tumours such as head and neck squamous cell carcinomas and breast cancer [48,49]. Interestingly, in breast cancer STAT5 seems to contribute to tumour initiation but it also promotes differentiation in established tumours and is predicted to be an indicator for favourable clinical outcome [50].

It must be stressed that STAT factors could be implicated at different stages in GIST. On one hand, they may contribute to the growth of the affected tumour cell during an early stage of tumour initiation but may then even become obsolete as other growth promoting mechanisms take over. On the other hand, and maybe much more importantly, STAT factors are known to have profound effects on the tumour microenvironment [13,14,51]. To our knowledge this aspect of GIST tumour biology has not yet been explored. Although STAT5 responses can also contribute to anti-tumour immunity, the effects of STAT3 and STAT1 on tumour-associated inflammation are believed to be playing a leading role. Whereas STAT1 is usually considered to be a tumour suppressor, the role of STAT3 is thought to be oncogenic [14]. Both STAT3 and STAT1 also regulate tumour angiogenesis and metastasis, albeit in opposite ways. Constitutive STAT3 activation within the tumour cell is transmitted to the tumour microenvironment where it induces tumour-associated inflammation. The tumour promoting effects of STAT3 affect both innate and adaptive immune responses and have made it an attractive target for cancer therapy. The balance between STAT1 and STAT3 activation within a tumour is paramount for the resulting effects on the tumour cell itself and the microenviroment [14]. Concerning STAT1 it has been proposed that an efficient STAT1 response requires the presence of activated STAT1/STAT1 homodimers [16] and that the efficient formation of this species can be influenced by the presence and the expression levels of STAT3 via the formation of STAT1/STAT3 heterodimers [16,52]. Thus, the respective levels of STAT1 and STAT3 can have profound effects on their biologic responses. Most interestingly, we found that the oncogenic PDGFRα mutant proteins lead to the formation of STAT1/STAT1 homodimers and accordingly we could detect the transcription of STAT1 specific target genes such as IRF1 (Figures 7 and 8). Somewhat surprisingly, most of the activated STAT3 seems to be present in STAT1/STAT3 heterodimers. Although little is known about the potency of this species concerning the induction of STAT3 regulated genes this finding could indicate that the panel of possible STAT3 responses are even not fully exploited in our PDGFRα mutant cell lines. We think that the formation of the STAT1 and STAT3 homo- and heterodimers is an extremely important parameter which needs to be considered when investigating the biological role of STAT factors in GIST. Dependent on the patient-specific background, differences in STAT1 and STAT3 levels, their specific activation mechanism as well as the extent of their phosphorylation may be decisive for generating either a tumour-promoting or a tumour-suppressive effect (or none of the two). In a patient-dependent manner, STAT factors may thus affect tumour cells or tumour-associated inflammation and play crucial roles for disease initiation and/or progression.

## Conclusions

To sum it up, we show that the overall oncogenic signature of the mutant kinases is thus the result of different signatures emanating from different cellular compartments (Figure 9) and that STAT mediated responses are an integral part of signalling via mutated PDGFRα proteins. Dissecting the physiological role of the different STATs in GIST is not a trivial matter as it is probably strongly influenced by the individual expression levels of the involved STATs as well as potential cross-talk events affecting the balance between the different STAT dimer species. However, in regard to potential personalised treatment of GIST patients this road is worth exploring.

## Methods

### Materials

Gleevec (Symansis) was dissolved in DMSO and supplemented for indicated time points. Recombinant human OSM (working concentration: 25 ng/ml) was obtained from Peprotech, recombinant human PDGFAA (working concentration: 250 ng/ml) was purchased from Immunotools. BrefeldinA was obtained from Sigma.

The expression plasmid for PDGFRα was generated based on the pLNCX2-PDGFRα plasmid generously provided by Prof. Andrius Kazlauskas (Boston). Point mutations were introduced using the Quik-change site-directed mutagenesis kit (Stratagene) following the manufacturer’s recommendations. The constructs were then transferred to pcDNA5/FRT/TO (Invitrogen) for the generation of stable cell lines. For the generation of pcDNA5/FRT/TO-PDGFRα-GFP expression plasmids, the coding sequences of the respective PDGFRα-wt or -mutant constructs were transferred into a pcDNA5/FRT/TO/GFP vector (C-terminal GPF-tag) using standard cloning techniques.

### Cells

The 293FR host cell line was generated from Hek293 cells by co-transfection of the Flp-In™ target site vector (pFRT/lac Zeo, Invitrogen) and Tetracyclin repressor (pcDNA™6/TR, invitrogen) using TransIT®-LT1 Transfection reagent (Mirus) according to manufacturer’s recommendations. Stable cell lines were selected and cultivated in presence of 10 μg/ml Blasticidin and 100 μg/ml Zeocin™ (Invitrogen). Primary normal human dermal fibroblast lines (NHDF) were generously provided by Prof. Jens M. Baron (RWTH-Aachen, Germany). The isolation of NHDFs from the donors was performed as described previously [53].

293FR cells were maintained in DMEM supplemented with 10% fetal bovine serum (FBS, PAA laboratories) in humidified atmosphere containing 5%CO_2_. For the FRT-recombinase reaction, a 7.5 fold surplus of Flp recombinase expression plasmid (pOG44, Invitogen) compared to the transgene expression plasmid (pcDNA5/FRT/TO-based, Invitrogen) was co-transfected. Stable transfected cells resulting from site directed recombination were selected and cultivated in presence of 100 μg/ml Hygromycin and 10 μg/ml Blasticidin. Experiments were conducted under serum reduced (1%) conditions for 11 h and for additional 3 h under serum free (0% FBS) conditions with 5 ng/ml doxycycline (Sigma) unless differently stated in the figure legends. Stimulation of PDGFRα wild-type expressing cells was performed with PDGF-AA (250 ng/ml) for the indicated times. For experiments involving BrefeldinA treatment in parallel to doxycycline treatment, the induction times were reduced to 4-6 h to avoid toxic effects of BrefeldinA.

### Cell lysis and Western blot analysis

All steps of cell lysis and immunoprecipitation were performed at 4°C using ice cold buffers. Cells were lysed on the dish with 1x Lämmli buffer. Proteins were subjected to SDS-PAGE, transferred to a polyvinylidene difluoride membrane (PALL) and probed with the respective antibodies. Primary antibodies against PLCγ and phosphospecific antibodies against STAT3 (pTyr705), ERK1/2 (pThr202/pTyr204), PDGFRα(pTyr849)/β(pTyr857), AKT (pSer473) and the HA-tag (6E2) were purchased from cell signaling. Anti-STAT1 and anti-STAT3 antibodies and phosphospecific antibodies for STAT1 (pTyr701), STAT5 (pTyr694) and PLCγ1 (pTyr783) were purchase from BD. Antibodies against STAT5 (C-17), PDGFRα (C-20), ERK1 (K-23), AKT1/2 (N-19) and tubulin (DM1A) were purchased from Santa Cruz Biotechnologies. Anti-GFP antibody was obtained from Rockland. The horseradish peroxidase-conjugated secondary antibodies were purchased from Dako (anti-goat Ab) or cell signalling (anti-rabbit and anti-mouse Abs). Signals were detected using an ECL solution containing 2.5 mM luminol, 2.6 mM hydrogenperoxide, 100 mM Tris/HCl pH 8.8 and 0.2 mM para-coumaric acid [54].

### Detergent-free preparation of nuclear extracts and Electrophoretic Mobility Shift Assay (EMSA)

The preparation of nuclear extracts and the EMSA using a mutated SIE oligonucleotide from the c-fos promotor were performed as previously described [55]. The EMSA using the casein oligonucleotide (β-casein(s): 5′-AGA TTT CTA GGA ATT CAA ATC-3′; (as) 5′-GAT TTG AAT TCC AAG AAA TCT-3′) was performed as for the SIE oligonucleotide ( m67SIE(s): 5′-GAT CCG GGA GGG ATT TAC GGG AAA TGC TG-3′; (as): 5′-AAT TCA GCA TTT CCC GTA AAT CCC TCC CG-3′) except that the oligonucleotide was radioactively labeled using the 5′ end-labelling procedure. For this 10 μl of casein oligonucleotide (100 pmol/μl; sequence) was incubated with 5 μl γ32 dATP (10 mM), 2 μl H_2_O, 2 μl buffer A (Fermentas/Thermo Scientific; 500 mM Tris/HCl, pH7.6, 100 mM MgCl_2_, 50 mM DTT, 1 mM spermidine) and 1 μl T4-polynucleotide kinase (10U/μl, Fermentas/Thermo Scientific) for 20 min at 37°C. Protein concentrations of nuclear extracts were measured using a NanoDrop spectrophotometer (PEQLAB). The DNA-bound STAT complexes were visualized using a Typhoon phosphorimager (Amersham Pharmacia).

### Fluorescence activated cell sorting (FACS)

Cell surface expression of PDGFRα and the GIST-mutants was analysed by flow cytometry using a FACS CantoII Instrument (Becton Dickinson, Heidelberg, Germany). Cells were harvested in presence of PBS/10 mM EDTA and washed with FACS-buffer (PBS/5%FCS/0.1%NaN_3_). Cells were incubated with 1.25 mg PDGFRα-primary antibody (anti-CD140A; 556001; BD Biosciences). Specificity was controlled using identical concentration of an isotype-matched antibody (IgG2a; 21335021; Immunotools). After washing, antibody binding was detected with a phycoerythrin-conjugated IgG-F(ab)_2_ fragment (Dianova). 1x10^5^ cells were analysed with a 488 nm argon laser light for excitation and a 576 nm longpass filter for detection.

### Microscopy

#### Live cell microscopy

293FR cells stably expressing PDGFR-wt-GFP or mutant-GFP proteins seeded onto poly-L-Lysine coated cover slips at least 24 h before induction with doxycycline. Induction of protein expression with doxycycline (5 ng/ml) was started 14 h before microscopy. Gleevec treatment (1 μM) was performed in parallel to doxycycline induction. Confocal live cell imaging (37°C, 5% CO2, Krebs-Ringer-Hepes medium + Glucose) was performed using a Zeiss LSM510 invert laser scanning microscope. GFP was excited with laser light (488 nm) and fluorescence was detected using a longpass Filter 505 nm (LP505).

#### Imaging of fixed specimens

293FR cells stably expressing PDGFR-wt or mutant proteins were cultured on poly-D-Lysine coated micro-slides (Ibidi, Germany). Protein expression was induced with doxycycline (5 ng/ml) for 14 hrs. Cells were fixed with paraformaldehyde 4% (Polysciences, Germany), permeabilized with Triton X-100 (0.1% in PBS) and blocked with 2% BSA before treatment with primary antibodies against PDGFRα (Abcam) and GM130 (BD Biosciences Europe). PDGFRα was subsequently labelled with Alexa Fluor 568 anti-rabbit antibody and the GM130 with Alexa Fluor 488 anti-mouse antibody (Molecular Probes). PDGFRα antibody specificity was checked using the PDGFR-negative Hek293 parental cell line. Imaging of the samples was performed on an Andor Revolution W1 spinning disc confocal microscope, mounted on a Nikon Ti microscope, using a 60x oil immersion objective (N.A. 1.49); laser lines: 405 nm, 488 nm and 561 nm; detector: Andor Neo SCMOS camera. For the detection, single band-pass filters were used (440/40 nm, 521/21 nm, 607/34 nm). Multi-color z-stacks were imaged using Andor iQ3.2 imaging software. Further data procession was done in FIJI (ImageJ) software. To allow the presentation of PM resident PDGFR and the Golgi resident protein GM130 in the same image, the required number of confocal slices were extracted from the recoded z-stacks and subjected to maximum projections.

### Microarray analysis

293FR cells inducibly expressing PDGFRα-wt, −V561D, −D842Y or -D842V mutant proteins (maintained in Dulbecco’s modified Eagle’s medium supplemented with 1% fetal calf serum) were treated with 5 ng/ml doxycycline for 11 h in order to induce the expression of the respective transgene. The medium was then switched to FCS free medium containing 5 ng/ml doxycycline for another 3 h. Cells expressing the wild-type PDGFRα were either stimulated with 250 ng/ml PDGF-AA for a total of 14 h (PDGFRα-wt(14 h)) or were left untreated (PDGFRα-wt(0 h)). The PDGFRα-wt(0 h) condition served as negative control for the stimulated wild-type receptor and for the oncogenic mutants. For microarray analysis, RNA of three biological replicates was isolated using the miRNeasy Mini Kit (Qiagen) according to manufacturer’s instructions with additional on-column DNase I digestion. RNA quality and purity was assessed using a 2100 Bioanalyzer (Agilent Technologies) and Nanodrop Spectrophotometer (Thermo Scientific), respectively. Gene Expression analysis was performed using GeneChip® Human Gene ST 1.0 arrays (Affymetrix).

The raw data in the form of Affymetrix CEL files was imported into Partek® Genomics Suite™ software (Partek GS) and the Robust Multichip Average (RMA) was applied to the data set [56]. Pre-adjustment for GC content with quantile normalisation and a mean probe set summarisation was used in this procedure as suggested by the default pipeline of Partek GS. All arrays were thus normalized to correct for systematic difference due to sample preparation. Only the core probe sets were considered for further analysis. The generated data set was then subject to rigorous quality control investigation, looking for the presence of outliers and further confounding effects. Principal Components Analysis (PCA) was applied in order to identify outliers and batch effects [57].

We focused on genes differentially expressed across the mutants comparing with PDGFRα-wt system. Differentially expressed genes were statistically evaluated by Partek® multi-way ANOVA, controlling for the batch effect due to scanning date. In order to correct for the false discovery rate (FDR), the Benjamini & Hochberg step-up method correction was applied [58]. Probe-sets with a step-up FDR <0.05 were considered to be significantly differentially expressed genes (SDEGs). As indicated in the figure legends most analyses were performed by additionally only considering SDEGs with an absolute fold change exceeding 40% (in comparison to non-stimulated PDGFRα-wt control cells).

Microarray data are available in the ArrayExpress database (www.ebi.ac.uk/arrayexpress) under accession number E-MTAB-2101.

### Rank-rank analysis

Most existing methods of comparing gene expression data sets require setting arbitrary cut-offs (e.g., fold changes or statistical significance), which could select genes according to different criteria [59,60]. In order to unambiguously compare the biologic response induced by PDGFRα mutants and wild-type, we used the nonparametric rank-rank hypergeometric overlap analysis (RRHO) [17]. This method allows the identification of statistically significant overlap or discrepancy between the gene signatures across the different mutants and normal signalling.

The probe sets were first ranked from the most significantly down-regulated to up-regulated ones. Signs of –log10 transformed ANOVA p-values were set concordant to the sign of fold change between F/PDGFRα or stimulated PDGFRα-wt and control (non-stimulated wild type PDGFRα). Then, the probe sets were sorted based on these signed values. The results of the analysis can be represented as a group of two plots: 1. The rank scatter plot represents the overlap between two signatures. Spearman rank correlation coefficient (rho) was calculated between the compared two gene signatures [61]. 2. RRHO heat map. The heat map value represents the –log10 transformed hypergeometric p-value [62] for the likelihood of observing the given overlapping number of genes between the two rank thresholds, visualized as pixel on the map (step size was set as default). The maximum of the heat map value can be used as an indicator for the strength of the observed overlap trend between two ranked gene lists [17]. We used the Benjamini-Yekutieli (BY) FDR correction for multiple hypothesis correction [63].

### Network analysis and visualization

In order to get more insight into the mutated and normal PDGFRα signalling we generated a merged signalling/transcriptomics gene regulatory network. Our goal was to build an integrated gene regulatory network based on differentially expressed genes and verified signalling components. The SDEGs with step-up FDR less than 0.05 and fold-changes greater than 40% (in comparison to non-stimulated PDGFRα-wt control cells) were uploaded into MetaCore®, which is a web-based computational platform primarily designed for the functional analysis of experimental data such as microarray data to identify regulatory networks and involved pathways (http://thomsonreuters.com/metacore/). We used the most stringent direct interaction (DI) algorithms to determine the relationship between the SDEGs “seeds” with high-confidence, manually-curated, peer-reviewed and cell-type specific interactions from the MetaCore® database (non-connected clusters and genes were removed). Later manual network curation was performed with Cytoscape [64].

From the original list of 595 coherently regulated SDEGs between the two mutants D842Y, V561D and the non-stimulated PDGFRα-wt control as well as the verified activated signalling components (PDGFRα, PLCγ, AKT, ERK1/2, p38, STAT1, STAT3 and STAT5), we obtained a *global PDGFRα-mutant regulatory network* consisting of 254 nodes and 873 function relations. A similar *PDGFRα-wt regulatory network* was constructed by involving only the SDEGs between PDGF-AA stimulated conditions (14 h) and non–stimulated control and the active conventional signalling (PDGFRα, PLCγ, AKT, ERK1/2), which resulted in a connected network of 61 node and 135 edges. An overlay of both the mutant gene regulatory network and the wild-type network is shown in Figure 7.

By applying the same pipeline we obtained an *oncogene-specific PDGFRα network* by including only the 463 onco-specific common SDEGs between the mutants (D842Y, V561D) and the non-stimulated PDGFRα-wt control and which are additionally not significantly altered in PDGF-AA stimulated conditions. These 463 SDEGs were integrated with the observed oncogenic signalling components (PDGFRα, PLCγ, AKT, ERK1/2, p38, STAT1, STAT3 and STAT5) to generate the network. This oncogene specific network was then further used for the generation of the strongly connected networks represented in Additional file 1: Figures S2 and S3.

### Strongly connected component (SCC) core network

Signalling components (e.g. proteins, RNAs, metabolites etc.) can influence other components by activation or inhibition and thus change their state in the network. Some of these changes may contribute to the network shifts from the healthy to the diseased stable state [65]. Hence it is important to identify such genes which are potentially determinant for the global network stability. An SCC is defined as a directed network of nodes for which a path exists from each node within the network to every other node. The hallmark of such an SCC is that it allows mutual information flow from one gene to any other within the structure, based on its specific connectivity [66]. Therefore, the state of any node in the SCC possesses the potential to directly or indirectly affect the state of any other node. This mutual influence between any pair of nodes within the SCC indicates that the SCC plays a crucial regulatory role relevant to network stability.

Based on the *global PDGFRα-mutant regulatory network* and on the *Oncogene-specific PDGFRα network* mentioned above, the strongly connected components in these networks were thus isolated using the Binom plugin [67] in Cytoscape [64]. Starting from the *global PDGFRα-mutant regulatory network* we obtained a unique strongly connected component (SCC) network consisting of 95 nodes within the PDGFRα-mutant gene regulatory network. This structure potentially determines the stability of the corresponding gene regulatory network. The network is visualised in Additional file 1: Figure S2. Furthermore, using the same method, we generated a strongly connected components network composed of 55 nodes for which we only considered the *Oncogene-specific PDGFRα network.*

## Additional file

Additional file 1:
**Supplementary Figures 1 to 3.**

## Abbreviations

BFA: BrefeldinA
Dox: Doxycycline
ERK: Extracellular signal regulated kinase
FDR: False discovery rate
PCA: Principal Components Analysis
PDGF: Platelet-derived growth factor
PI3K: Phosphatidyl-inositol-3-kinase
PLC: Phospholipase C
MAPK: Mitogen activated protein kinase
MEN: Minimal essential network
OSM: Oncostatin M
RRHO: Rank-rank hypergeometric overlap
RRSP: Rank-rank scatter plot
SDEG: Significantly differentially expressed genes
STAT: Signal transducer and activator of transcription
RTK: Receptor tyrosine kinase
TKI: Tyrosine kinase inhibitor.
