# Supplementary material for: Constitutive activation of oncogenic PDGFRα-mutant proteins occurring in GIST patients induces receptor mislocalisation and alters PDGFRα signalling characteristics
Source: Cell Commun Signal. 2015 Mar 31;13:21. doi: 10.1186/s12964-015-0096-8 (PMC4396151; doi:10.1186/s12964-015-0096-8)
Supplement: Additional file 1: — Supplementary Figures 1 to 3. [file 12964_2015_96_MOESM1_ESM.pdf]

Supplementary figure 1

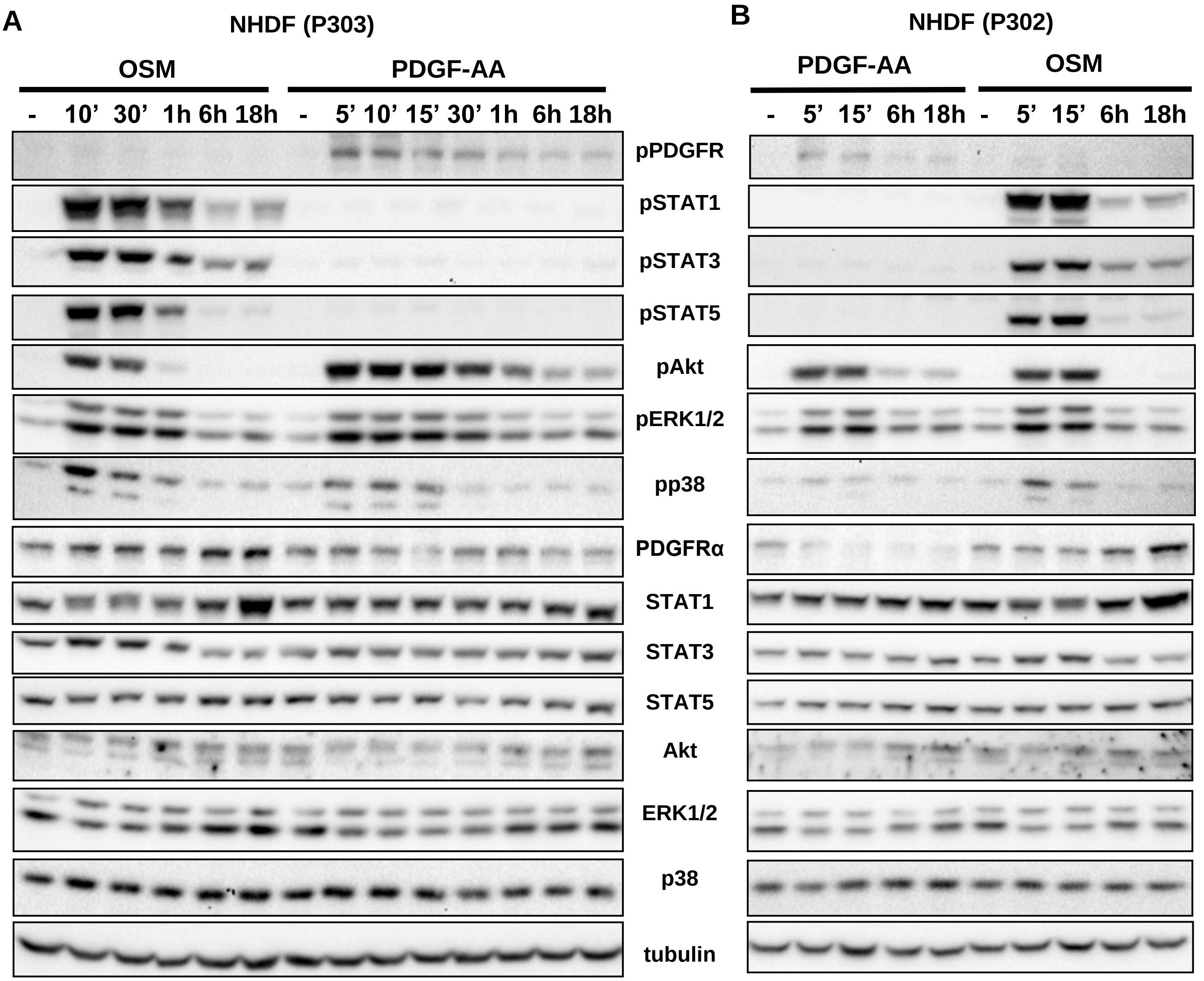

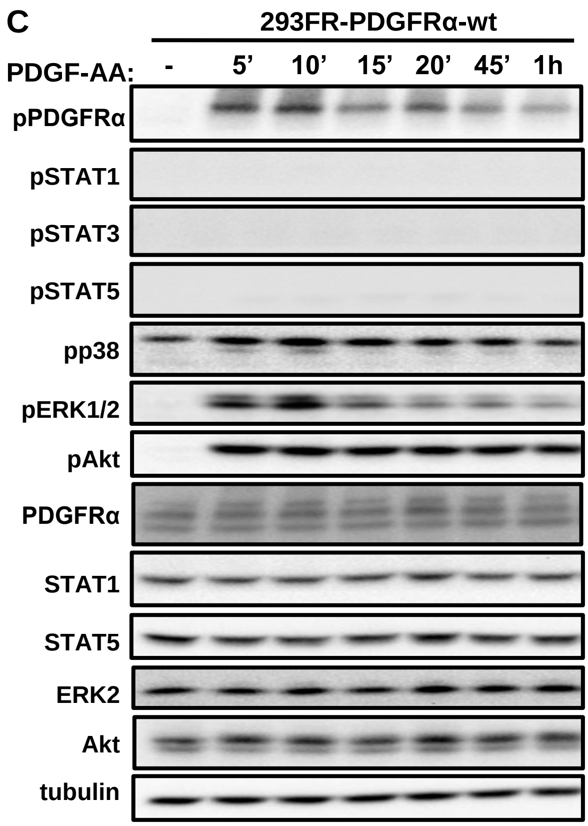

Signalling caracteristics of PDGF-AA

(A, B) NHDF (P303 and P302) cells were treated with 20 ng/ml OSM or 20 ng/ml PDGF-AA for the times indicated and phosphorylation of PDGFR, STAT1, STAT3, STAT5, AKT and ERK and p38 was monitored by Western blot analysis. A tubulin stain is provided as control. **(C)** 293FR-PDGFRα cell lines stably expressing PDGFR-wt were treated with doxycycline for 18h and additionally treated with PDGFR-AA for the indicated times. Activation and expression of PDGFRα, STAT1, STAT3, STAT5, AKT, ERK1/2 and p38 was assessed by Western blot analysis. A tubulin stain is provided as control.

Supplementary figure 2

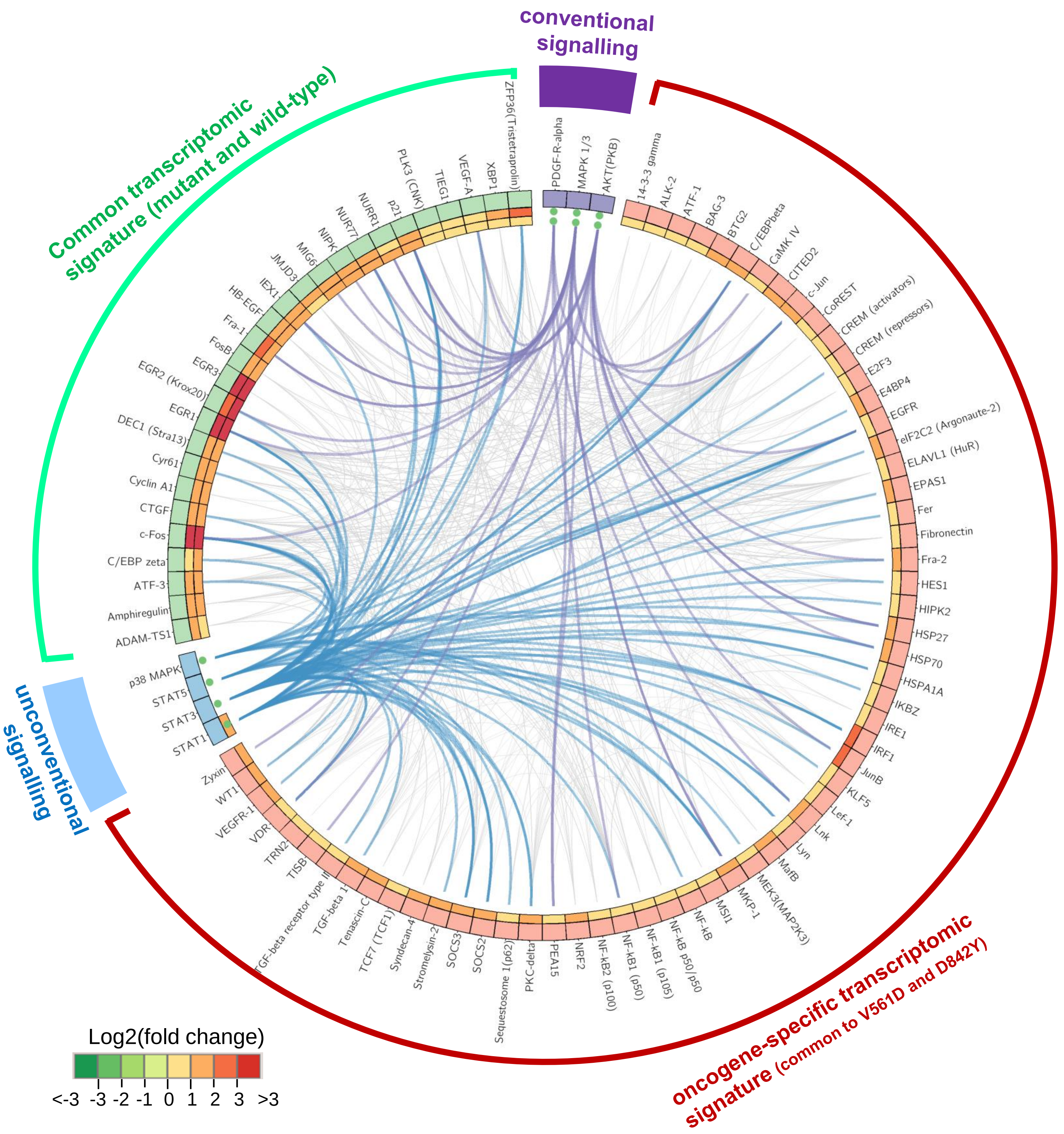

Strongly connected components network based on the *global PDGFRα-mutant regulatory network*. Circos plot representing the SCC network for the common signalling and transcriptomic signatures of the V561D and D842Y mutant proteins. Only SDEGs with a step-up FDR less than 0.05 and absolute fold change exceeding 40% (in comparison to non-stimulated PDGFRα-wt control cells) are represented. The SDEGs were divided into two groups: 1) the common regulated genes between the oncogenic situation and the PDGF-AA stimulated wild-type protein (highlighted in green), 2) SDEGs which are exclusively regulated under the oncogenic situation (red). The average log2 transformed fold change is represented as heat maps (outer heat map circle: merged V561D/D842Y response; inner heat map circle: PDGF-AA stimulated receptor). The observed signalling characteristics are represented as conventional (violet) and unconventional (blue) signalling. The activation of these signalling components by the mutant or the wild-type receptors is indicated by green dots. The interactions between the molecules in the networks were visualized as violet (conventional signalling to transcriptomic responses), blue (unconventional signalling to transcriptomic responses) or grey (transcriptomic to transcriptomic) connections.

Supplementary figure 3

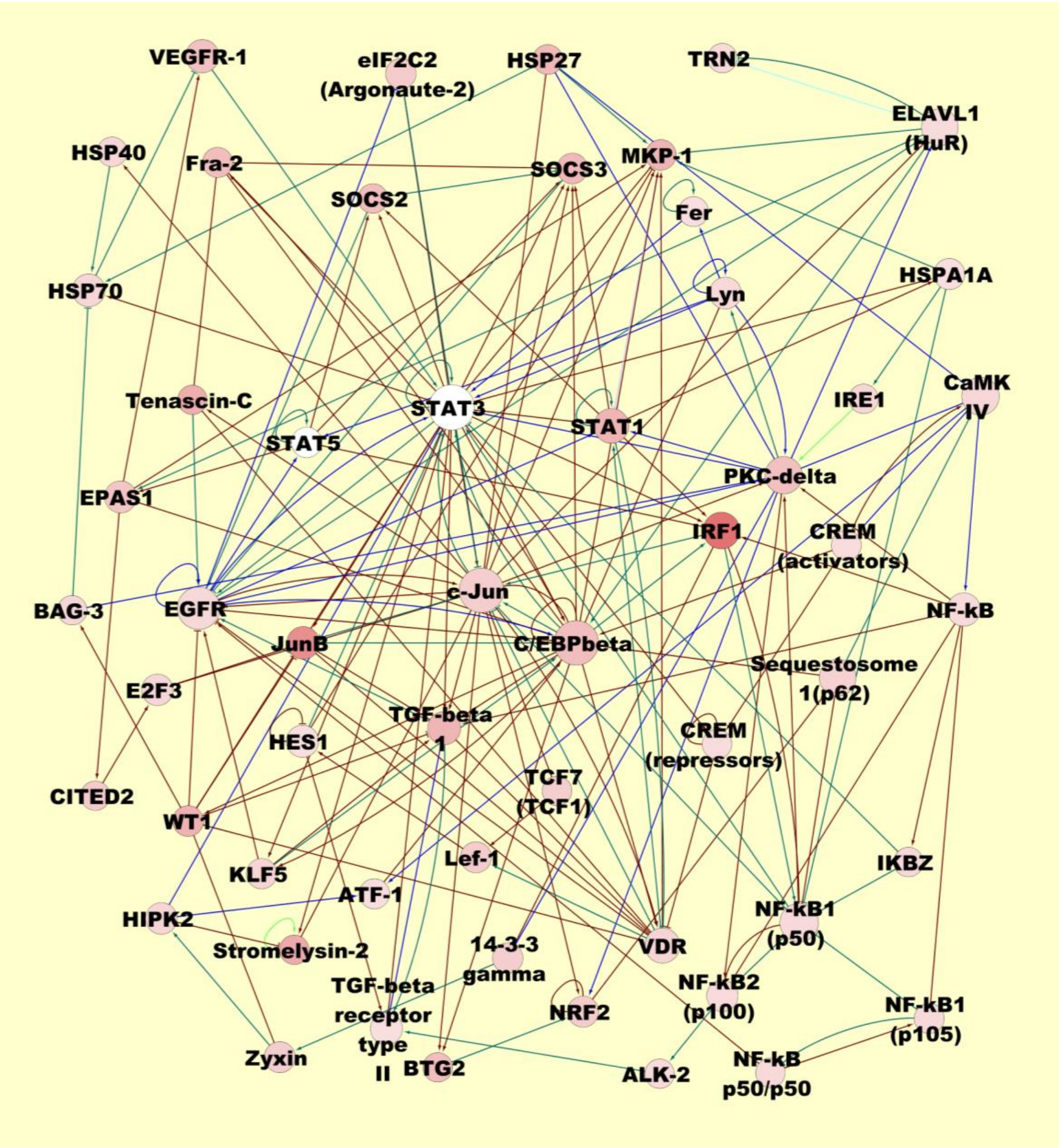

Mapping Type	Discrete Mapping
Binding	
Cleavage	
Dephosphorylation	
Deubiquitination	
Phosphorylation	
Transcription regulation	
miRNA binding	

Strongly connected components network based on the *Oncogene-specific PDGFRα network*. SCC network representation for the common signalling and transcriptomic signatures of the V561D and D842Y mutants. Only oncogene-specific SDEGs with a step-up FDR less than 0.05 and absolute fold change exceeding 40% (in comparison to non-stimulated PDGFRα-wt control cells) have been considered. The extent of up-regulation of the different genes is indicated by the intensity of the red colour. Network components which do not meet the set SDEG criteria (FDR < 0.05 and absolute fold change > 40%) are represented with a white background. The nature of the interactions is indicated by the colour of the connections and the color code is given below the figure.
